# Physicochemical and Release Characteristics of Andrographolide Liquisolid Systems Fabricated Based on Microcrystalline Cellulose, Magnesium Aluminometasilicate, and Their Blends

**DOI:** 10.3390/polym18212667

**Published:** 2026-10-28

**Authors:** Napaphak Jaipakdee, Peera Tabboon, Pinpair Ekkanjanakorn, Kattiya Sonchiangkham, Thaned Pongjanyakul, Ekapol Limpongsa

**Affiliations:** 1 Faculty of Pharmaceutical Sciences, Khon Kaen University, Khon Kaen 40002, Thailand; jsaowanee@kku.ac.th (N.J.); peerata@kku.ac.th (P.T.); pinpair.e@kkumail.com (P.E.); kattiya.s@kkumail.com (K.S.); thaned@kku.ac.th (T.P.); 2 Center for Research and Development of Herbal Health Products, Khon Kaen University, Khon Kaen 40002, Thailand; 3 College of Pharmacy, Rangsit University, Pathum Thani 12000, Thailand

**Keywords:** microcrystalline cellulose, magnesium aluminometasilicate, andrographolide, liquisolid carriers, Neusilin US2

## Abstract

This study aimed to investigate physicochemical properties and release characteristics of andrographolide liquisolid systems developed using microcrystalline cellulose (MCC), magnesium aluminometasilicate (NEU), and their combinations (MCC:NEU) as carriers. Polyoxyl 15 hydroxystearate combined with N-methyl-2-pyrrolidone as a non-volatile liquid vehicle facilitated AG solubility, leading to the transformation into non-crystalline AG in the liquisolid powder, as demonstrated via PXRD. NEU-based liquisolid powder exhibited a greater liquid-loading factor than that of MCC (0.925 vs. 0.202). Liquid-loading factor of MCC:NEU exhibited a notable improvement, resulting in an increase in AG loading from 1.63% to 4.20% as the NEU content increased from 0% to 75%. MCC:NEU-based liquisolid powders exhibited greater AG release and tabletability than MCC-based powders. Five prepared AG liquisolid tablets based on liquisolid powder, sodium starch glycolate (4%), and magnesium stearate (1%) had acceptable tablet properties and AG content uniformity. MCC-based liquisolid tablets showed a modest improvement in AG release with a release efficiency of 44.6 ± 2.5%, while NEU-based tablets had a release efficiency of 8.7 ± 0.7%. MCC:NEU-based AG liquisolid tablets exhibited increased disintegration time and thus significantly suppressed AG release. This suggests that a justification for the design of MCC:NEU liquisolid tablets with prompt disintegration is essential for achieving superior AG release.

## 1. Introduction

Liquisolid technology is acknowledged as one of the most promising, straightforward, and economical techniques to enhance the dissolution or release rate and bioavailability of weakly water-soluble pharmaceuticals, showing significant potential for industrial applications. Liquisolid systems are dry, non-sticky, adequately flowable, and compressible powdered formulations of liquid medications. Liquisolid technology facilitates the transformation of hydrophobic, poorly water-soluble compounds into solutions or suspensions (liquid medications), using an appropriate non-volatile liquid vehicle, which are subsequently converted into flowable powders by simple blending with chosen powder excipients, referred to as the carrier and coating materials [1,2]. To date, the liquisolid approach has effectively enhanced the in vitro release/dissolution and, thus, the in vivo bioavailability of poorly water-soluble active pharmaceutical ingredients (API) [3,4,5] and phytochemicals [6,7,8]. Solid excipients characterized by porous surfaces and elevated adsorption capabilities have been utilized as adsorbents/absorbents, featuring large particles with inherently good flowability as carriers, alongside fine nanometer-scale particles serving as coating materials [9].

Liquisolid technology has been shown to significantly enhance the release and oral absorption of numerous BCS class II API [1,3,4,5,10,11,12]. In recent years, the advancement of liquisolid systems has been extended to purify hydrophobic phytochemicals, including curcumin and quercetin [13,14], and andrographolide [7], as well as herbal crude extracts, including walnut extract [15], *Cannabis sativa* extract [10], *Caesalpinia pulcherrima* flower extract [16], and *Andrographis paniculata* (*A. paniculata*) extract [6]. The markedly enhanced biopharmaceutical characteristics, including release, permeability, or absorption, of these phytochemicals and phytopharmaceuticals have been evidenced [6,13,15,16,17]. The effectiveness of a liquisolid system in improving release depends extensively on its essential components, namely, the liquid vehicle, carriers, and coating materials. Each component fulfills a distinct role in enhancing the surface area and augmenting the release rate of the API [1]. A non-volatile liquid vehicle with high solvency capacity for the API is typically employed to achieve thorough molecular dispersion of the compound in the liquid and to preserve this dispersion throughout storage [6]. The carrier and coating materials function as porous substrates that adsorb/absorb the liquid medications, transforming wet mixtures into compressible, free-flowing powders. Carriers are regarded as the predominant portion contributing to the structural framework essential for the flowability and compressibility of the liquisolid powder [9]. Microcrystalline cellulose (MCC) is a refined, partially depolymerized form of cellulose consisting of approximately 150–400 glucose units connected by β-1,4-glucosidic linkages, produced via acid hydrolysis of cellulose fibers sourced from plants. In contrast to raw cellulose, MCC has a significant level of crystallinity and is characterized by white or almost white porous particles, generally in a short, rod-like, or powdered form. MCC has become an essential substance for numerous applications in the pharmaceutical sector owing to its distinctive characteristics, including renewability, biocompatibility, and mechanical robustness [18,19,20]. MCC is the most commonly used and initially adopted carrier in liquisolid formulations. It can be regarded as the first generation of the commonly utilized carrier in liquisolid technology. It offers outstanding compactability and flow characteristics essential for direct tablet compression. As a hydrophilic carrier that enhances wetting and swift disintegration, MCC may expedite the release of hydrophobic active ingredients [1,21]. However, due to its limited specific surface area (SSA) of approximately 1.18 m^2^/g, MCC-based liquisolid systems have restricted flowable liquid retention capacity, constraining liquid medicament loading. Their liquid load factor ranged from 0.195 to 0.302 [9,22,23,24]. This resulted in an excessively large and unmanageable dosage, particularly for phytochemicals that typically require substantial doses [25,26].

The SSA of carriers is a crucial attribute that affects the absorptive properties of liquisolid systems, facilitating increased liquid medication loading and, thus, a higher dosage of the active ingredient. To enhance the threshold of liquid loading, modern carriers with substantial SSA and elevated absorption capabilities, such as anhydrous dibasic calcium phosphate and magnesium aluminometasilicate, are employed [27,28]. Magnesium aluminometasilicate, or Neusilin^®^ US2 (NEU), is one of the mesoporous materials that possesses a significant SSA and has become the major carrier in liquisolid systems. NEU is a water-insoluble, amorphous Al_2_O_3_·MgO·1.7SiO_2_·xH_2_O in spherical particle form with a significant mesoporosity and an SSA of approximately 300 m^2^ per gram. NEU maintains satisfactory flow characteristics and tabletability, even when incorporating high ratios of liquid medications [6,9,29,30]. Previous studies have indicated that NEU is a carrier with the capacity to absorb the highest volume of liquid medication, approximately 110–125% of its mass, while maintaining excellent flowability of the liquisolid mixtures [9]. A recent study indicated that among the three mesoporous silica-based carriers examined (Syloid^®^ XDP 3050, Syloid^®^ XDP 3150, and NEU), NEU demonstrated a superior capacity to incorporate greater quantities of the liquid phase, achieving a liquid load factor of up to 1.3, while preserving acceptable flowability and tensile strength in comparison to the other two carriers [28]. Owing to its superior SSA, the use of NEU as a carrier has been indicated to render coating materials unnecessary, thereby potentially simplifying the manufacturing of liquisolid systems [31]. In recent years, NEU has emerged as a prominent carrier in liquisolid systems [3,6,9,28,31,32,33,34,35,36]. Nonetheless, its mesoporous architecture makes it an effective adsorbent, which may result in incomplete desorption of API [3,33,37,38,39,40,41]. NEU is an exceptionally specialized spray-dried raw material that is more expensive than MCC. Moreover, owing to its extremely porous and naturally lightweight characteristics, which result in a low bulk density (significant bulkiness), the manipulation of NEU-based powder can lead to substantial weight variations and inadequate filling in high-velocity tablet press dies, attributable to suboptimal volumetric flow and air entrapment. Furthermore, NEU possesses limitations owing to its inherent inability to swell in aqueous environments, and disintegrating NEU-based tablets are significantly dependent on an auxiliary agent for disintegration [33].

The vast majority of liquisolid system investigations rely on neat MCC or NEU as carriers. The restricted application of their blended systems signifies a notable research gap, particularly because their combination may provide unique synergistic benefits. This study aimed to investigate the physicochemical and release characteristics of the model phytochemical, andrographolide (AG), from various liquisolid systems fabricated using MCC, NEU, and NEU:MCC mixtures as carriers. The particular MCC:NEU ratios (75:25, 50:50, and 25:75) were chosen to systematically investigate the performance of liquisolid powders and tablets based on the MCC-rich (75:25), equal (50:50), and NEU-rich (25:75) blends compared with neat MCC and NEU. Notably, assessing this comprehensive spectrum is crucial for providing fundamental details on the characteristics of the resultant liquisolid systems of this binary excipient. The formulations of AG liquisolid tablets (AGLST) were developed using liquisolid powder, a superdisintegrant (4% sodium starch glycolate), and a lubricant (1% magnesium stearate). The physicochemical and release characteristics, along with the tabletability and disintegrability of the liquisolid powder, were also investigated for comparison purposes. Molecular interactions were studied using FTIR spectroscopy, and solid-state characterization was also performed using PXRD.

AG is a versatile diterpene lactone derived from *A. paniculata* and is characterized by the chemical formula C_20_H_30_O_5_. It exhibits extensive pharmacological properties, including anti-inflammatory, antioxidant, anticancer, antibacterial, antihyperglycemic, neuroprotective, and immunoregulatory activities. Its pharmacological effects have been corroborated by findings from several in vitro and in vivo experimental frameworks, including human and animal cell lines and animal models [42,43]. AG is recognized for its inadequate release behavior and low bioavailability, attributed to its limited aqueous solubility (0.05 mg/mL [6]), and is categorized as a Biopharmaceutical Classification System (BCS) class II substance. This property is a significant impediment to its clinical use [42,44,45]. Additionally, the poor oral bioavailability of AG is due to its metabolism and efflux by P-glycoprotein (P-gp) [46]. Various pharmaceutical formulation techniques, such as solid dispersions [47], nano/micro-formulations [48,49,50], and liquisolid systems [6,7], have been effectively utilized to improve the release and oral bioavailability of AG. Liu et al. [7] indicated that liquisolid powders markedly improved the oral bioavailability of the AG, with relative bioavailability enhancements of approximately 312% and 1.37-fold compared to raw AG material and commercial AG tablets (guttate pills), respectively. In accordance with this formulation, AG was dissolved in a combination of N-methylpyrrolidone (NMP), polyethylene glycol 6000, and Soluplus as the liquid medium and then adsorbed onto porous starch as the carrier adsorbent to create liquisolid powders. Recent findings suggest that the liquisolid powder of *A. paniculata* crude extract utilizing neat NMP as a liquid vehicle and NEU-Aerosil^®^ 200 (Aerosil) as a carrier-coating material markedly enhanced oral bioavailability by as much as 4.9 times [6]. The formulations of *A. paniculata* crude extract in the liquisolid powder form not only improved AG release but also diminished the source-dependent variability in AG release [51]. According to the aforementioned study, neat NMP served as a liquid vehicle, whereas MCC-Aerosil functioned as a carrier-coating material system. To date, the characteristics of the liquisolid systems of AG in the form of tablets/compacts have not been documented. Compressing liquisolid powders into compact tablets can notably influence their release behavior by decreasing their porosity and surface area.

Polyoxyl 15 hydroxystearate or Kolliphor HS 15 (KHS) was used as a liquid vehicle in combination with NMP. NMP has been reported to yield the greatest solubility of AG and has been successfully used to prepare a liquisolid powder of *A. paniculata* crude extract [6,51]. KHS is a non-ionic surfactant primarily composed of polyethylene glycol-15-hydroxystearate, which is known for its potential to inhibit P-gp [52]. Using a P-gp inhibitor excipient may benefit liquisolid systems of P-gp substrates such as AG. A previous investigation asserted that the enhancement of oral absorption and bioavailability of AG from AG liquisolid powder may be associated with the presence of Soluplus, which could act as a P-gp inhibitor [7]. Notably, the usage of KHS as a binary solvent mixture with NMP for the solubility of AG has not been reported.

Colloidal silicon dioxide or Aerosil was employed as a coating material at a coating material-to-carrier proportion of 1:20. Aerosil comprises exquisite nanoscale particles, featuring an extensive SSA of approximately 200 m^2^/g. This nanoscale enables Aerosil to envelop carrier particles and absorb surplus liquid, enhancing system dryness and powder flowability. Furthermore, an optimum quantity of Aerosil may enhance the flow by reducing interparticle friction and preventing aggregation [30].

## 2. Materials and Methods

### 2.1. Materials

Andrographolide (AG, ≥98% C_20_H_30_O_5_) was sourced from Weifang Wehibest Supply Chain Co., Ltd. (Weifang, China). The reference standard AG was supplied by the Bureau of Drug and Narcotic Department of Medical Sciences, Ministry of Public Health (Nonthaburi, Thailand). N-methyl-2-pyrrolidone (NMP) was sourced from QRëC (Auckland, New Zealand). Polyoxyl 15 hydroxystearate (USP/NF) (Kolliphor^®^ HS 15, KHS) was supplied by BASF SE (Ludwigshafen, Germany). Magnesium aluminometasilicate (Neusilin^®^ US2, NEU) was procured from Fuji Chemical Industries Co., Ltd. (Osaka-shi, Osaka, Japan). Microcrystalline cellulose (MCC, Avicel^®^ PH102) and colloidal silicon dioxide (Aerosil^®^200, Aerosil) were obtained from Onimax Co., Ltd. (Bangkok, Thailand). Hydrochloric acid (HCl) was supplied by RCI Labscan Co., Ltd. (Bangkok, Thailand). Ethyl alcohol (EtOH, L Pure^®^ 99.8) was sourced from the Liquor Distillery Organization (Chachoengsao, Thailand). Sodium starch glycolate (Explotab^®^, JRS Pharma) was supplied by Rama Production, Co., Ltd. (Bangkok, Thailand). Magnesium stearate was purchased from Mallinckrodt Pharmaceuticals (St. Louis, MO, USA). HPLC-grade methanol and acetonitrile were sourced from Fisher^®^ Scientific (Loughborough, UK). All chemical reagents were of analytical grade and obtained from commercial suppliers.

### 2.2. Solubility of AG

The solubility of AG in KHS and KHS:NMP mixtures (KHS:NMP ratios (*w*/*w*) of 2:1, 1:1, and 1:2) was assessed by combining 0.5 g of AG powder with 1.0 g of the solvent in capped vials. The resulting mixture was subjected to ultrasonic stimulation for 1.5 h and then kept in a thermostatic shaking water bath for 36 h at 30 ± 1 °C. The acquired mixture was filtered through a 0.45 μm nylon membrane syringe filter (Agilent Technologies, Inc., Santa Clara, CA, USA) and diluted with methanol. The resultant filtrate was examined for AG content using HPLC.

### 2.3. Preparation of AG Liquisolid Powder

Five liquisolid powders of AG (AGLS) with different carrier types were formulated using KHS:NMP at a 1:2 (*w*/*w*) ratio as the liquid vehicle (Table 1). The liquid load factor for each carrier-coating system was derived from calculations using the flowable liquid retention potential (Φ-value). The Φ-value measurements of the neat carriers (Φ_MCC_ and Φ_NEU_) and coating substances (Φ_Aerosil_) were established using the angle of slide method [53,54]. A fixed mass (3 g) of each solid substance was pulverized with 1:2 KHS:NMP, increasing by 0.1 g for each addition. After each addition, the angle of slide of the mixture was assessed using the inclined plane technique with a stainless-steel apparatus. A diagram depicting the relationship between the ratio of 1:2 KHS:NMP to the solid mass and the associated angle of slide was created. The proportion resulting in an angle of slide of 33° was determined to be the Φ-value for 1:2 KHS:NMP. The Φ-values of the MCC:NEU mixes (Φ_MCC:NEU_) were obtained using calculations utilizing the Φ-values and their respective weight fractions (W) of the neat carriers in the mixture (Equation (1)). The liquid load factor metrics for all the carrier-coating substances were computed using Equation (2).

(1) $$\Phi_{\mathrm{MCC}:\mathrm{NEU}}=\left(\Phi_{MCC}\cdot W_{MCC}\right)+\left(\Phi_{NEU}\cdot W_{NEU}\right)$$

(2) $$Liquid\;load\;factor=\Phi_{Carrier}+\Phi_{Aerosil}\cdot 1/R$$

where Φ_carrier_ and Φ_Aerosil_ denote the flowable liquid retention potential of the carrier and Aerosil (coating materials), respectively. R refers to 20, which is the carrier-to-coating ratio used in this study.

To prepare the AGLS, the necessary amount of AG was measured and subsequently mixed with a specified amount of 1:2 KHS:NMP. The concentration of AG in the liquid medication (AG-1:2 KHS:NMP solution) was maintained at 10% *w*/*w*. The mixture was subjected to ultrasonication for 60 min. The resulting solution was subsequently combined with a carrier and then Aerosil powder, utilizing a glass mortar and pestle for 5 min each. The physical mixture of AG (AGPM) with 50:50 NEU:MCC or MCC was also prepared as a control. The necessary amount of AG was accurately measured and uniformly combined with the carrier and coating using a rotomixer for 3–5 min. All the produced powders were maintained in a securely sealed, light-resistant container and stored in refrigerated conditions.

### 2.4. Characterization of AGLSs and AGPMs

#### 2.4.1. Flowability

The flowability characteristics were evaluated using the Carr index and the angle of slide. The Carr index was determined using the bulk and tapped densities of the powders, using the method adopted from the conventional methodology outlined in the USP. A precise measurement of ten grams of the test mixture was taken and placed in a 100 mL cylinder. The bulk volume was recorded to evaluate the bulk density of the sample. Subsequently, the cylinder holding the powder was tapped 400 times, and the tapped density was determined using the tapped volume. The Carr index was calculated using the equation outlined elsewhere [55].

The angle of slide was determined using the angle of slide tester. A powder sample weighing 1 g was positioned at the end of the inclined plane of the slide tester. The upper section of this plane was gradually raised until the powder began to move. The angles created by the plane plate and the horizontal surface were recorded.

#### 2.4.2. Fourier Transform Infrared Spectroscopy (FTIR)

FTIR spectra for AGLSs (AGLS-100N, AGLS-25M75N, AGLS-50M50N, and AGLS-100M), blank liquisolid powders (B-LS-100N and B-LS-100M), physical mixture of AG with blank liquisolid powder ((AG)(B-LS-100N)-PM and (AG)(B-LS-100M)-PM), along with AG, AG solution in KHS, and 1:2 KHS:NMP, as well as KHS and 1:2 KHS:NMP, were obtained using the TENSOR27 FTIR spectrometer (Bruker Optics GmbH & Co. KG, Ettlingen, Germany) coupled with an attenuated total reflection (ATR) accessory. A modest quantity of each specimen was placed on the ATR plate section. The spectrum across a region of 4000–600 cm^−1^ was recorded using a resolution of 4 cm^−1^ and consisted of 32 consecutive scans at a controlled room temperature of 23 ± 2 °C.

#### 2.4.3. Powder X-Ray Diffraction (PXRD)

PXRD was conducted utilizing a powder X-ray diffractometer (Rigaku MiniFlex, Rigaku Holdings Corporation, Tokyo, Japan) featuring a Cu Kα source. The analysis was performed at 40 kV and 15 mA, with a Bragg angle 2θ varying between 5–40° and a step angle of 0.02° (2θ)/s.

#### 2.4.4. Analysis of AG Content

The amounts of AG in the AGLSs or AGPMs, equivalent to 3 mg of AG, were weighed and dispersed in EtOH (100 mL). The combination was ultrasonicated for 45 min and magnetically stirred for 16 h. After filtration, the resulting solution was analyzed for AG content using HPLC.

#### 2.4.5. In Vitro Release

The release behaviors under non-sink conditions of AG from AGLSs and AGPMs were evaluated using a double-walled jacketed glassware with 100 mL of release medium: simulated gastric fluid (SGF, 0.1 M hydrochloric acid medium, pH 1.2) or simulated intestinal fluid (SIF, pH 6.8 phosphate buffer). The release medium hydrodynamics were generated using a magnetic stirrer (WH-610D, Wiggens GmbH, Wuppertal, Germany) operating at a stirring rate of 200 rpm, with the temperature maintained at 37 ± 0.5 °C (WiseCircu WCB-6 model; Daihan Scientific Co., Ltd., Seoul, Republic of Korea). The test powder equivalent to 5 mg of AG was added to the medium. At specified intervals, an aliquot of the medium (1 mL) was withdrawn and replaced with pre-heated medium. After filtration, the resulting sample was analyzed for AG content using HPLC. To accurately ascertain the cumulative release percentage of AG over time, modifications for the sequential extraction and substitution of 1 mL release samples were implemented, and the mass of AG extracted during each sampling interval was considered. A plot illustrating the accumulation of AG release percentage with time was generated. The percentage of AG released at 30 min (Q_30min_, %) and the release efficiency (RE, %) were determined using Equation (3). The similarity of the AG release in SIF compared with that in SGF was assessed using the similarity factor (f_2_, Equation (4)).

(3) $$Release\;efficiency,\;RE=\frac{\int_{0}^{t}y\times dt}{y_{100}\times t}$$

(4) $$f_{2}=50\times log\left[\frac{1}{\sqrt{1+\left(\frac{1}{n}\right)\sum_{t=1}^{n}{\left(R_{t}-T_{t}\right)}^{2}}}\times 100\right]$$

where t refers to time. n denotes the number of sampling points, and R_t_ and T_t_ indicate the AG release (%) in SGF (references) and in SIF (tests), respectively.

#### 2.4.6. Tabletability and Disintegration Ability

Tableting of the AGLSs and AGPMs was performed using a hydraulic press (Powder Hydraulic Pressing-LITH-15T, Xiamen Lith Machine Limited, Xiamen, China) coupled with a 10 mm diameter flat-faced circular mold. A predetermined amount of the tested powder (300 ± 5 mg) was manually placed into a die. Compression pressures of 1.25, 2.5, and 5.0 MPa were applied without holding time. The resulting compacts were analyzed for thickness using a vernier caliper (Mitutoyo Corporation, Kawasaki, Japan). The tensile strength (MPa) was derived from the crushing force (F, N), determined using a hardness tester (Model 40–2100 VK200, VanKel^®^, Cary, NC, USA), and calculated using Equation (5).

(5) Tensile strength = 2F/πDh

where D and h are the diameter (mm) and thickness (mm) of the tablet, respectively.

The disintegration ability of the compact compressed with a 1.25 MPa force was determined utilizing a basket-rack assembly disintegration test apparatus (Model ZT-324, Erweka America Inc., Edison, NJ, USA) with 0.1 M HCl (1000 mL, 37 °C). The time at which the compact disintegrated completely was recorded [55].

### 2.5. Preparation of AG Tablets

The AG tablets containing 15 mg of AG, based on the AGLS or AGPM, were prepared. Briefly, the AGLS or AGPM (50 g) was blended with 2 g of sodium starch glycolate for 3 min and then with 0.5 g of magnesium stearate for 1 min. Subsequently, the resulting powder blend was compacted using a Powder Hydraulic Pressing-LITH-15T machine (Powder Hydraulic Pressing-LITH-15T, Xiamen Lith Machine Limited, Xiamen, China) coupled with a 13 mm diameter circular mold. The compression pressure that produced the tablet with a hardness within the range of 60 ± 10 N was used. For AGLST-100N, AGLST-75N25M, AGLST-50N50M, and AGLST-25N75M, a compression load of 1.25 MPa with a 30 s holding time was used. In the case of AGLST-100M, AGPMT-100N, and AGPMT-50N50M, a compression load of 1.0 MPa with a 30 s holding time was used. The resultant AGLSTs and AGPMTs were collected and stored in a sealed container for future evaluation.

### 2.6. Characterization of AG Tablets

#### 2.6.1. Tablet Properties

Weight variation was assessed by individually weighing 20 random tablets using an analytical balance (Joan Lab Equipment Co., Ltd., Huzhou City, China). The thickness of 20 random tablets was assessed using a vernier caliper (Mitutoyo Corporation, Kawasaki, Japan). The hardness was assessed using a VK200 VanKel hardness tester (Model 40–2100 VK200, VanKel^®^, Cary, NC, USA). The friability of tablets (≥6.5 g total weight) was assessed using a VanKel friabilator (Model 45-2200, VanKel^®^, Cary, NC, USA).

The disintegration time was determined using a disintegration test apparatus (Model ZT-324, Erweka America Inc., Edison, NJ, USA). A total of 1000 mL of 0.1 M HCl, maintained at a temperature of 37 °C, was used as the disintegration medium. The time at which the tablet fully disintegrated was recorded [55].

#### 2.6.2. Determination of AG Content

Each AG tablet was accurately weighed, meticulously crushed, and subsequently transferred to flasks containing 100 mL of EtOH. The mixture underwent sonication for 45 min and was stirred for 16 h. Following filtration through a 0.45 μm membrane and appropriate dilutions, the samples underwent analysis via HPLC assay.

#### 2.6.3. Determination of AG Release Behavior

The release behavior under non-sink conditions of AG was evaluated using a paddle apparatus (Erweka dissolution tester, Langen, Germany). 600 mL of SGF, kept at 37 ± 0.5 °C, was used as the release medium. The rotation speed was maintained at 50 ± 2 rpm. The test AG tablet was placed directly into the release vessel assembly of the apparatus. Aliquots of the release medium (10 mL) were extracted and replaced with new medium at designated time intervals within 2 h. The concentration of AG was determined using HPLC. To determine the cumulative release percentage of AG over time, justification for the sequential extraction and substitution of 10 mL release samples was implemented, and the mass of AG extracted during each sampling interval was considered. A graphic representation of the cumulative release percentage with time was generated. The release parameters, including Q_30min_ and RE, were determined.

### 2.7. HPLC Analysis

AG was measured using the validated HPLC methodology reported previously [51,56]. An LC-2050 series Shimadzu (Kyoto, Japan) HPLC apparatus, coupled with a diode array detector set at 254 nm and a C18 reversed-phase column (Eclipse Plus, 4.6 × 150 mm, 3.5 µm, Agilent Technologies, Inc., USA), was used. Elution was carried out with a binary solvent system of 0.1% formic acid in acetonitrile (A) and a solution of 0.1% formic acid in water (B) as the mobile phase at a flow rate of 1.4 mL/min, and the temperature was set to 20 °C. The gradient program followed this protocol: an isocratic phase at 30% A was maintained from 0 to 3 min, and the gradient then increased to 40% A between 3 and 6 min. From 6 to 9 min, a stable phase at 40% A was maintained. The gradient was elevated to 80% A between 9 and 10 min, and an isocratic phase at 80% A was sustained from 10 to 12 min. Between 12 and 13 min, the gradient was reduced to 30% A, and from 13 to 15 min, an isocratic phase at 30% A was maintained. A 20 µL injection volume was used. The AG peak exhibited distinct separation with a retention time of 4.2 min [56]. The HPLC methodology has been shown to exhibit remarkable linearity within the tested concentration range of 3.125–50.0 µg/mL (R^2^ > 0.9999). The method was precise, with intra-day and inter-day %RSD values below 3.97%, and accurate, with an accuracy ranging from 95.8% to 105.7%. The limits of detection (LODs) and limits of quantification (LOQs) were established at 0.14 and 0.45 µg/mL, respectively. The validation outcomes adhered to the AOAC International guidelines for analytical method validation [51].

### 2.8. Statistical Evaluation

Results are expressed as an average accompanied by the standard deviation (SD) of the technical replicates, the individual specimens (powders or tablets) sampled from a single representative batch of each formulation, to evaluate the within-batch pharmaceutical quality and uniformity. This technical replication approach, adopted from USP 40–NF 35 guidelines [55], is commonly employed in solid dosage form studies. Where specified, a one-way ANOVA, accompanied by post hoc Tukey testing (SPSS (Thailand) Co., Ltd. (Bangkok, Thailand)), was conducted to evaluate the statistical significance among the formulations. A significance threshold of *p* < 0.05 was employed.

## 3. Results and Discussion

### 3.1. AG Solubility

**Figure 1:**
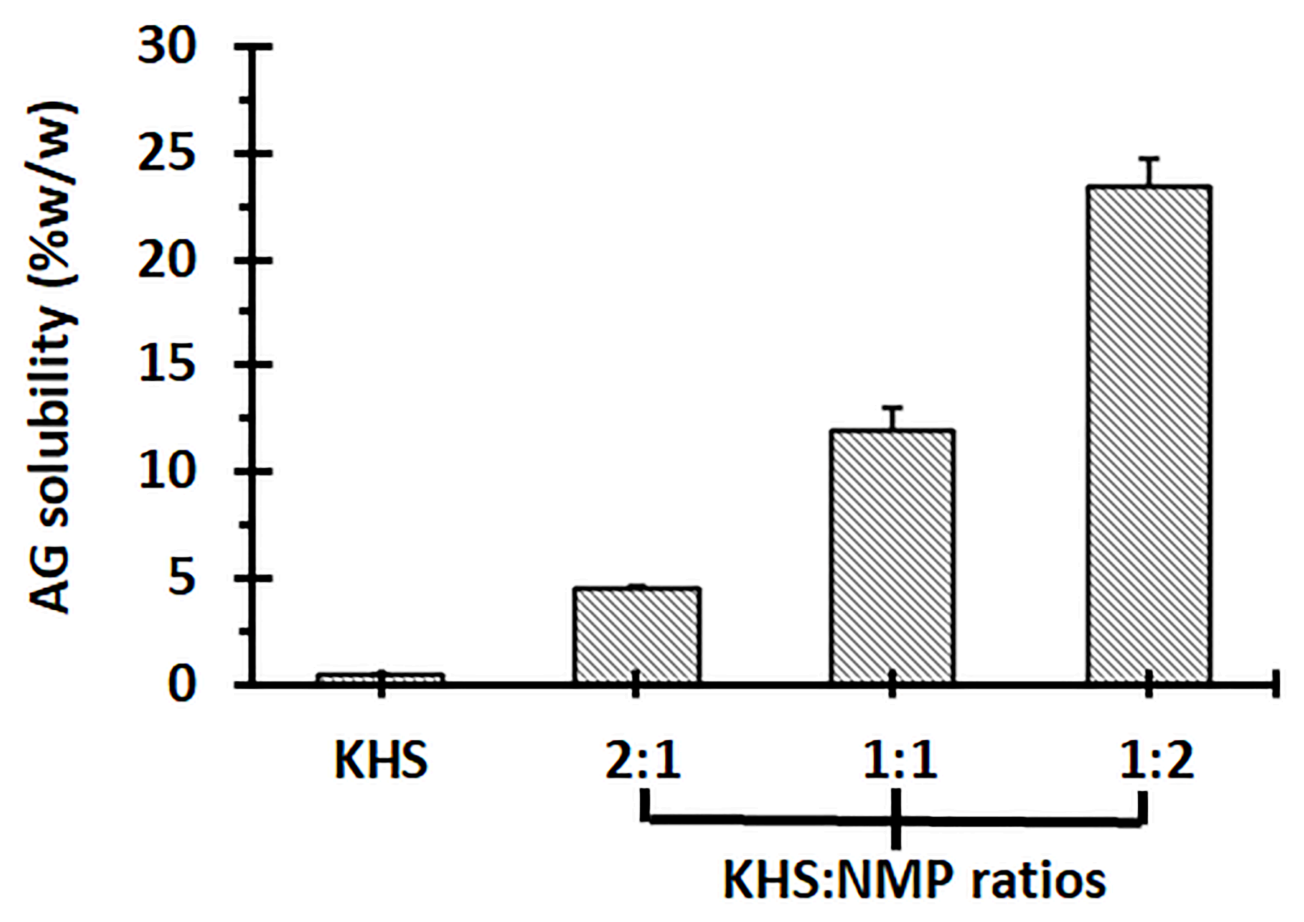
Solubility values (30 °C) of AG in KHS and KHS:NMP mixtures (2:1, 1:1, and 1:2 KHS:NMP ratios (*w*/*w*)). Data are presented as mean ± SD (*n* = 3).

The solubility of AG in the KHS and KHS:NMP mixture was determined, and the results are illustrated in Figure 1. KHS was a poor solvent for AG, with a solubility value of only 0.46 ± 0.01% *w*/*w*. This aligns with other findings regarding the solubility of AG in nonionic surfactant liquids, such as sorbitan monooleate, caprylocaproyl macrogolglycerides, polyoxyl 35 castor oil, and polysorbates [51,57]. The incorporation of NMP significantly improved AG solubility in the KHS-based liquid, and the values increased proportionally with the NMP ratio in the KHS:NMP mixture. The AG solubility value reached 23.5 ± 1.3% when a 1:2 KHS:NMP mixture was used. These results confirm the superior solvency capacity of NMP. These findings underscore AG’s limited solubility and indicate that a greater proportion of NMP in the binary solvent system is essential for effective solubilization.

It should be noted that among the non-volatile liquid vehicles investigated, NMP was the only solvent that provided sufficient AG solubility, achieving up to 38% *w*/*w* (30 °C) [6,7,51]. Previous research indicates that AG demonstrates inadequate solubility (37 °C) in various fixed oils, oleic acid, and α-tocopherol, along with nonionic surfactants including Tweens, Span 80, and Cremophore EL, with a solubility of <3.5 mg/mL. Similarly, low-polarity solvents, such as glycerin, propylene glycol, polyethylene glycol 400, and Transcutol HP, exhibited a solubility value ≤ 54 mg/mL (37 °C) [50,57,58]. NMP is a non-volatile liquid that functions as an exceptionally potent solvent and solubilizing agent in many formulations, including injectable, oral, and topical formulations [30,59]. Various NMP-based drug delivery platforms, including liquisolid and self-nanoemulsifying drug delivery systems, have been developed to enhance the solubility and oral bioavailability of poorly water-soluble substances [6,7,60,61,62]. NMP functions as a solvent in a commercially available in situ implant composed of a biodegradable polymer within NMP, specifically the Atrigel Delivery System, at a concentration of approximately 50–65% [59]. The administration of the Atrigel Delivery System for leuprolide acetate via subcutaneous injection, referred to as Eligard, resulted in NMP exposure levels ranging from 160 to 258.5 mg per dose, depending on the dosage of leuprolide acetate [63]. Notably, NMP is not listed in the FDA Inactive Ingredient Database (IID). Despite NMP exhibiting minimal acute toxicity in animal studies upon oral administration and NMP-based liquisolid systems showing oral LD_50_ values surpassing 5 g/kg [6,64], the suitable application of NMP as an oral excipient in pharmaceutical formulations faces regulatory challenges owing to its significant reproductive and developmental toxicity. In experiments on rats examining intergenerational reproductive toxicity, the No-Observed-Adverse-Effect Level (NOAEL) for alterations in pup body weight was established at 160 mg/kg/day [65]. Consequently, NMP is classified as an ICH Q3C Class 2 solvent in medicinal formulations, with a permissible daily exposure threshold of 5.30 mg/day [59,66].

Owing to its capacity to address the solvation requirements for AG, a KHS:NMP ratio of 1:2 was chosen as the model liquid vehicle for the liquisolid system of the model phytochemical, with a primary focus on examining the physicochemical characteristics and release behavior of various liquisolid systems formulated with MCC, NEU, and NEU:MCC as the carriers.

### 3.2. AGLS Powder Characteristics

#### 3.2.1. Physical Characteristics, Flowability and AG Loading

According to the solubility data, a 1:2 KHS:NMP mixture was the liquid vehicle demonstrating the greatest solubility for AG, which was expected to enhance rapid release and was consequently selected for the liquisolid formulation. Five AGLS powders were produced with MCC, NEU, or MCC:NEU combinations as carriers and Aerosil as the coating substance, utilizing the established liquid load factor calculated from the Φ-value. The Φ_MCC_, Φ_NEU_, and Φ_Aerosil_ measurements for 1:2 KHS:NMP were noted as 0.165, 0.888, and 0.738, respectively (Figure S1, Supplementary Materials). The Φ_MCC:NEU_ values of the MCC:NEU combinations were calculated from the weight contribution and Φ-value of their individual components using Equation 1. This equation was developed based on the assumption that no interactions occur between the carrier particles. Consequently, the Φ_MCC:NEU_ values for the MCC:NEU mixtures were found to be 0.313, 0.527, and 0.707 for the 75:25, 50:50, and 25:75 MCC:NEU mixtures, respectively. The greater Φ-values of NEU and Aerosil in comparison to MCC are associated with their accessible SSA, which are around 300, 200, and 1.18 m^2^ per gram, respectively [22,30,67]. Consequently, the MCC:NEU blends with a higher proportion of NEU exhibited elevated Φ-values. The Φ-value represents the greatest mass of liquid that a unit mass of powder material can accommodate while maintaining acceptable flow characteristics. This parameter serves as a crucial parameter in pharmaceutical mathematical models for the formulation of dry, free-flowing liquisolid powders [53,54].

**Figure 2:**
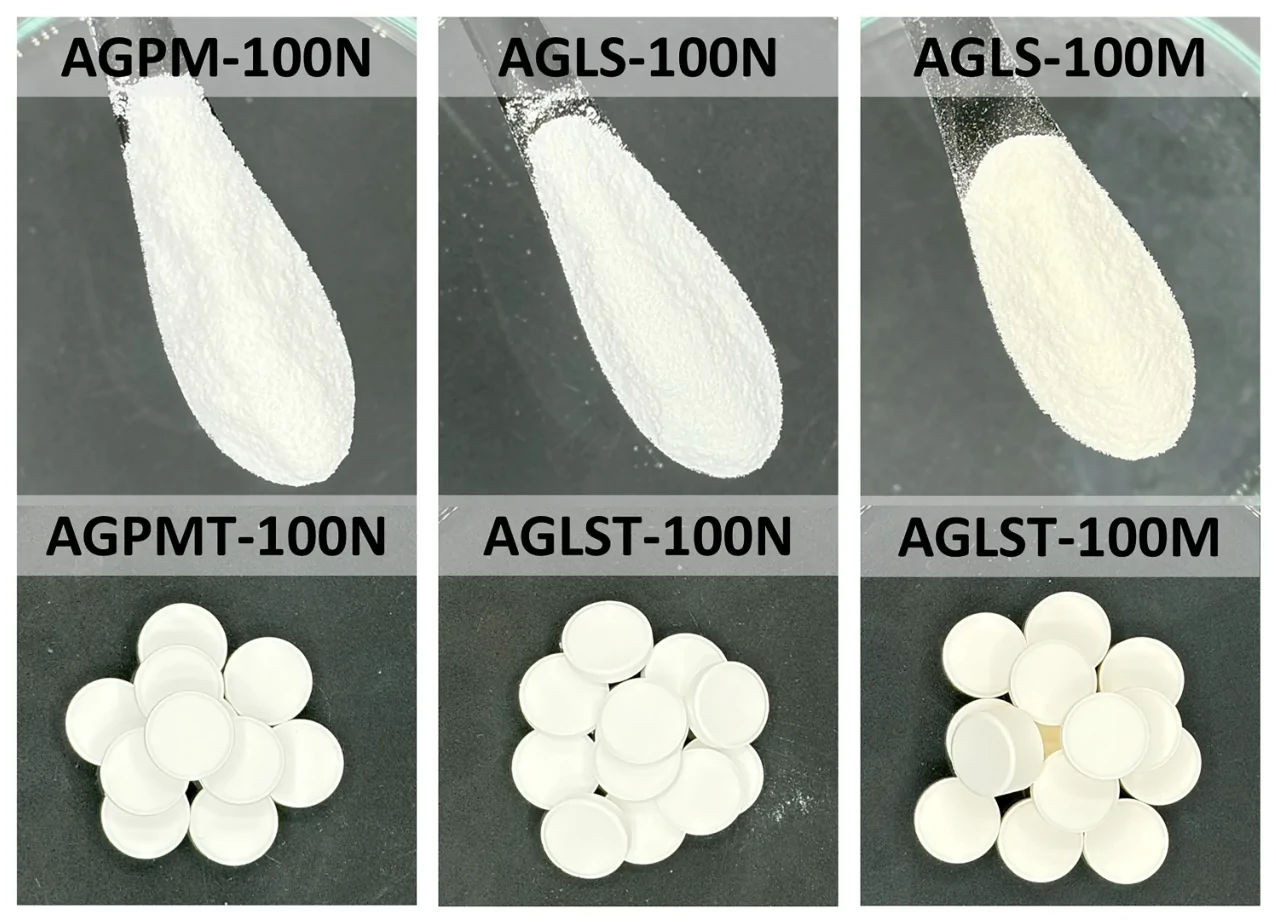
Physical appearance of AGPM and AGPMT, as well as AGLSs and AGLSTs.

Utilizing the Φ_carrier_ and Φ_Aerosil_ metrics, the liquid load factor for different carrier–Aerosil mixtures with a carrier-to-coating material ratio of 20 varied between 0.925 and 0.202 (Table 1). An increased Φ-value resulted in a heightened ability to load AG in 1:2 KHS:NMP solution, and thus, the AG loading in the powder formulations. All resulting AGLS powders were dry, non-hygroscopic, and uniform, with characteristics similar to AGPM (Figure 2, with the original images provided in Figure S3, Supplementary Materials). The flow characteristics of the AGLS and AGPM were assessed using the angle of slide and Carr index. Diminished values of these variables signify a lesser extent of interparticle friction and powder cohesiveness, resulting in enhanced flowability. Table 2 illustrates that the angle of slide for all evaluated powders was below 23.7, signifying their satisfactory flow characteristics. Studies have indicated that an angle of slide of 33° is the upper limit for the acceptable flow characteristics of liquisolid systems [15,32]. The angle of slide is a specific metric employed to evaluate the flow properties of formulated liquisolid powder blends [53,54].

Notably, in this study, the Φ-value of a binary carrier mixture was predicted from the weighted contributions and Φ-values of its individual components (Equation (1)) for the first time. The acceptable flowability of the MCC:NEU-based AGLS, which was formulated according to the liquid load factor obtained from these predicted Φ_MCC:NEU_ values (Equation (1)), verified the validity of this predictive calculation for the Φ-value of a binary carrier blend.

The AGLSs prepared using different carriers exhibited different bulk densities. Owing to the highly porous, amorphous spherical particles of NEU, AGPM-100N exhibited the lowest bulk density of 0.15 g/mL. The bulk density of AGPM-100N was determined to be within the reported bulk density range of magnesium aluminometasilicate material (0.13–0.18 g/mL [30]). The increased bulk density of AGPM-50M50N was associated with the higher bulk density of MCC (0.35 g/mL [30,68]). Compared with the respective AGPM, the formulation into AGLS resulted in a significant increase in the bulk density. This pertains to the incorporation of liquid medication (10% AG in 1:2 KHS:NMP solution) that absorbs and/or fills the empty spaces and pores inside the carrier particles, leading to a decrease in porosity and total pore volume [9].

The AGLSs demonstrated a Carr index below 16.6, signifying fair to good flow properties [55]. Significantly, AGPM exhibited a higher Carr index than its respective AGLS. This could be associated with the effect of incorporating AG powder into the blend. The fine hydrophobic properties of AG powder may enhance interparticle friction, foster cohesiveness, and/or induce electrostatic charging [69]. The acceptable flowability of AGLSs and AGPMs was primarily determined by the intrinsic flowability of the carrier material, which accounted for 50.64–90.5%. It is widely acknowledged that the flowability of powders improves with an increase in particle size and sphericity. The carrier particles, characterized by a greater average particle size of approximately 100 µm (NEU and MCC) and spherical morphology (NEU), provide good to excellent powder flow [54,70]. Aerosil, at 2.53–4.50%, served as a glidant, enhancing the mixture flowability by reducing friction among discrete particles [30]. Owing to their superior flowability characteristics, blending NEU with MCC did not adversely affect the flow properties of the powder mixture.

The acceptable flowability of the AGLS formulations indicated that the concentration of the 1:2 KHS:NMP solution did not exceed the retention capability of the carrier-Aerosil system, validating the appropriate liquid load factor employed in each formulation. An excessive quantity of 1:2 KHS:NMP solution would facilitate the development of liquid bridges, thereby enhancing capillary and van der Waals interactions.

The AGLSs demonstrated AG loading concentrations between 1.63% and 4.64%. This aligns with 99.11% to 101.86% of the theoretical load, exhibiting an SD of under 1.56% (Table 2). The disparity in AG loading between AGLS formulations was contingent upon the liquid load capacity of each carrier-Aerosil system. The AG loading of the MCC-rich-based AGLS was lower than that of formulations with a greater NEU ratio. The minimal SD value of AGLSs indicated a favorable uniform distribution of AG within the formulations related to the dissolving of AG in 1:2 KHS:NMP before its application over the carrier-Aerosil system. The percentage of AG labeling in the AGPM samples varied from 99.59 ± 3.72 to 101.08 ± 6.95%. The considerable standard deviation of AGPM indicated the variability of the AG in the sample.

Significantly, the AG content in the AG-1:2 KHS:NMP solution was maintained at 10% across all AGLS formulations. This concentration was chosen to guarantee complete dissolution of AG in the used liquid medication. Considering the AG solubility measurement in a 1:2 KHS:NMP solution (S_AG_, 23.5%, Figure 1) and its concentration in the AG-1:2 KHS:NMP solution (C_AG_, 10%), the fraction of molecularly dispersed AG (FM = S_AG_/C_AG_) would translate to 1 [23,71]. This suggests that all the incorporated AG can exist essentially in a molecularly dissolved form within the 1:2 KHS:NMP solution, thereby characterizing the liquisolid system. Prior studies have demonstrated evident correlations between FM values of 1 and the improvement of release, intestinal permeability, oral bioavailability, and the solid-state stability of the incorporated active ingredients [23,67,72].

#### 3.2.2. Compatibility Study

**Figure 3:**
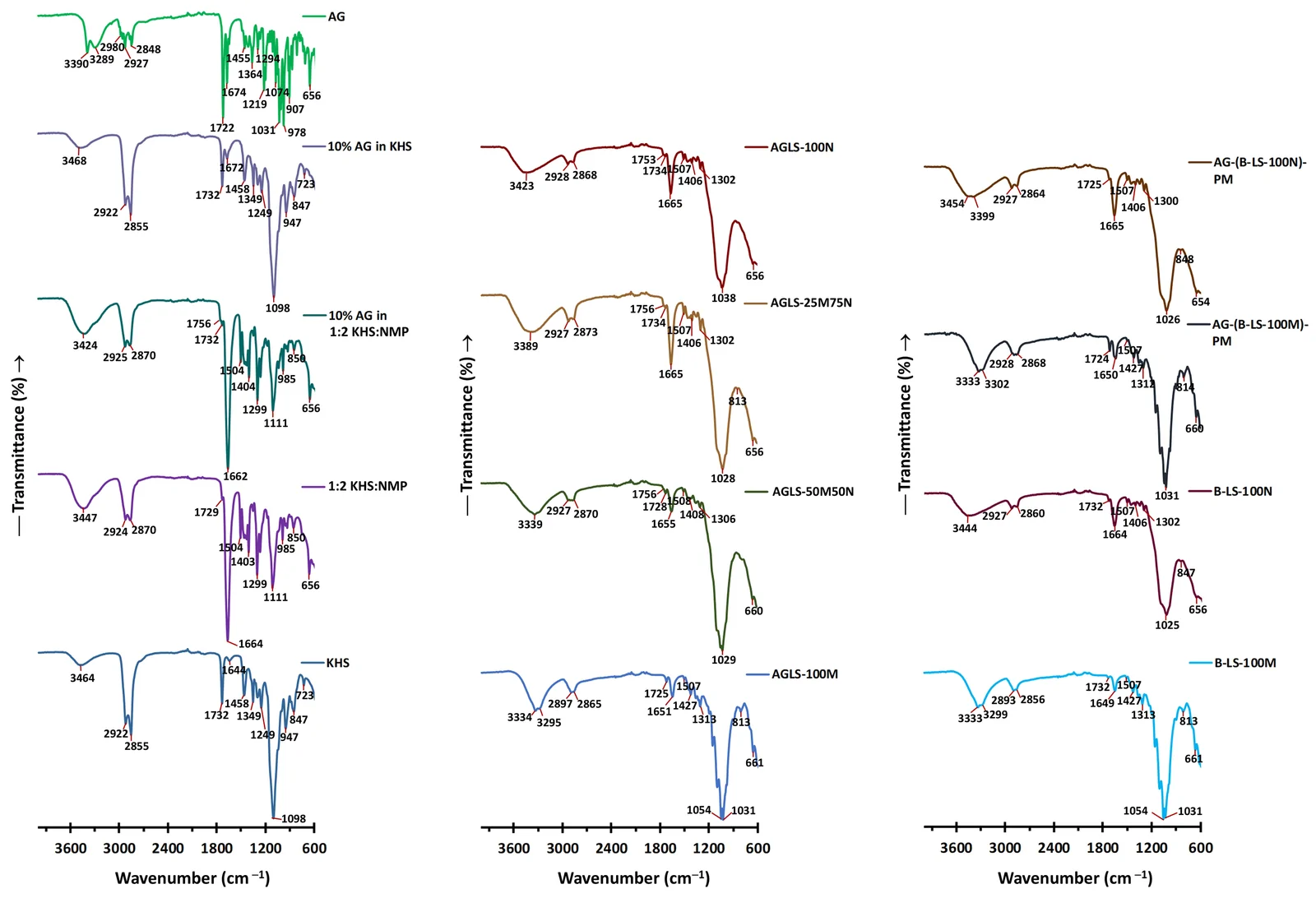
ATR-FTIR patterns of AG, AG solution in KHS, neat KHS, AG solution in 1:2 KHS:NMP, 1:2 KHS:NMP mixture, AGLSs (AGLS-100N, AGLS-25M75N, AGLS-50M50N, and AGLS-100M), physical mixtures of AG with blank liquisolid powder ((AG)(B-LS-100N)-PM and (AG)(B-LS-100M)-PM), and blank liquisolid powder (B-LS-100N and B-LS-100M).

The compatibility of AG with the liquisolid component was investigated using ATR-FTIR. Analysis of the FTIR data (Figure 3 and Table S2 (Supplementary Materials)) indicated that AG exhibited hydroxyl (O−H) stretching peaks at 3289 cm^−1^ and 3390 cm^−1^, suggesting an intermolecular hydrogen-bonded configuration in AG. The spectral bands identified between 2848 and 2980 cm^−1^ are associated with the stretching vibrations of aliphatic C–H bonds. The peaks at 1722 cm^−1^ and 1674 cm^−1^ signify the C=O stretching vibration of the lactone carbonyl moiety and the C=C stretching vibration associated with alkene groups, respectively. C−O−C stretching was detected at 1030 cm^−1^ and 1074 cm^−1^ for primary and secondary alcohols, respectively, while the out-of-plane bending of =CH_2_ or terminal alkene features appeared at 907 cm^−1^. The unique FTIR absorption peaks for KHS include a broad O–H peak near 3464 cm^−1^, an aliphatic C–H stretch ranging from 2855 to 2922 cm^−1^, a prominent ester C=O stretching at 1732 cm^−1^, and a distinct ether C–O–C stretch at 1098 cm^−1^. The infrared spectra of KHS corresponded to the recorded observations [73,74]. The combination of KHS and NMP (1:2 KHS:NMP solution) influences their FTIR peak positions, notably causing the O–H peak to shift to 3447 cm^−1^, while the C=O stretching of NMP shifts from 1670 cm^−1^ [6,51] to 1664 cm^−1^. This indicates hydrogen interactions between the carbonyl oxygen (C=O) of NMP and the hydroxyl groups on the stearate chain and terminal PEG chains of KHS. Reports indicate that the shift, disappearance, or broadening of characteristic FTIR peaks, particularly in the hydroxyl or carbonyl regions, in pharmaceutical combinations directly suggests physical or chemical changes in the molecular structure or bonding of a blend. The shift in both hydroxyl and carbonyl groups may be influenced by hydrogen bonding [11,12,75]. Previous studies have linked the shifts in the carboxylic group of griseofulvin and the hydroxyl group of the liquid vehicle (Cremophor EL) [12], as well as the reduced intensity, shifts, or disappearance of the C=O stretching region of spironolactone in the IR spectra of liquisolid formulations [11], to the formation of hydrogen bonding.

The FTIR spectra of the AG solution in KHS exhibited distinctive bands for both AG and KHS, suggesting no significant interaction between the two. In contrast, the FTIR spectra of the AG solution in 1:2 KHS:NMP exhibited the O–H peak shifting from 3447 to 3424 cm^−1^, while the C=O stretching vibration of the lactone carbonyl moiety shifted to 1756 cm^−1^. This suggests possible interaction, probably hydrogen bonding, between AG and the NMP-based solvent. AG features three –OH groups and a lactone carbonyl group [76] that could form hydrogen bonding and/or dipole–dipole interactions. This is consistent with a previous FTIR investigation of AG in neat NMP solution [6,51]. This hydrogen bond, considered a solute-solvent interaction, accounts for AG’s significantly higher solubility in 1:2 KHS:NMP than in KHS [11,12].

The blank liquisolid powder based purely on NEU (B-LS-100N), which included only 1:2 KHS:NMP and NEU-Aerosil, displayed distinct absorption peaks indicative of the functional groups present in 1:2 KHS:NMP, alongside NEU-Aerosil (O–H stretching approximately 3000–3600 cm^−1^ and Si–O stretching at approximately 950–1100 cm^−1^). Nevertheless, the B-LS-100N’s FTIR appearance, which indicated a –C=O group of NMP at 1664 cm^−1^ and –OH peaks at 3444 cm^−1^, may also be related to the molecular interaction between NMP and NEU. A previous report found a shift in the –C=O band of NMP from 1670 to 1665 cm^−1^, accompanied by the –OH peaks shifting to a higher wavenumber in the NMP-containing NEU liquisolid powder [6]. The capacity for hydrogen bonding of silanols in local environments on silica surfaces has been observed previously. Prior studies have indicated that silanol groups (Si–O–H) on the NEU surface can engage in hydrogen bonding with APIs, such as ibuprofen [77] and piroxicam [78].

The blank liquisolid powder composed of MCC (B-LS-100M) demonstrated a loss of the 1:2 KHS:NMP attributes, particularly those associated with NMP. It primarily exhibits absorption peaks indicative of the functional groups present in MCC, including O–H stretching (3333–3299 cm^−1^), C–H stretching (2893–2856 cm^−1^), H–O–H bending of adsorbed water molecules (1649 cm^−1^), CH_2_ bending (1427 cm^−1^), and C–OH stretching (1054 cm^−1^), in addition to the stretching peak of Si–O–Si from Aerosil at 813 cm^−1^ and the ester C=O stretching of KHS at 1732 cm^−1^. The absence of NMP features could be ascribed to the low levels of NMP loading (Table 1), which are below the ATR detection threshold.

The FTIR spectra of the physical mixture of AG and blank liquisolid powder ((AG)(B-LS-100N)-PM and (AG)(B-LS-100M)-PM) exhibited no alteration in the FTIR characteristics of either AG or blank liquisolid powder, indicating a lack of interaction among the components when they are physically combined. The FTIR spectra of AGLS-100N displayed the peak features of the AG solution in 1:2 KHS:NMP and NEU-Aerosil. The AGLS fabricated with a mixture of NEU and MCC exhibited the FTIR characteristics of AGLS-100N and AGLS-100M, depending on the NEU-to-MCC ratio. This may signify the physical deposition of the AG-1:2 KHS:NMP solution onto the NEU-Aerosil surface. In contrast, the FTIR spectra of AGLS-100M revealed the absence of peaks related to AG and NMP from the AG-1:2 KHS:NMP solution at 1656 cm^−1^ and 1662 cm^−1^, respectively. This could be ascribed to the diminished levels of AG and NMP loading (Table 1), which are below the ATR detection threshold.

It is important to recognize that chemisorption, including hydrogen bonding and physical entrapment or capillary retention between a liquid medication, such as an AG-1:2 KHS:NMP solution and a carrier surface, may influence the extent of desorption of the liquid into adjacent liquids. Typically, physical entrapment facilitates swift desorption because the surrounding medium readily displaces the non-volatile solvent, liberating the solubilized active component into the medium more rapidly.

#### 3.2.3. Solid State

**Figure 4:**
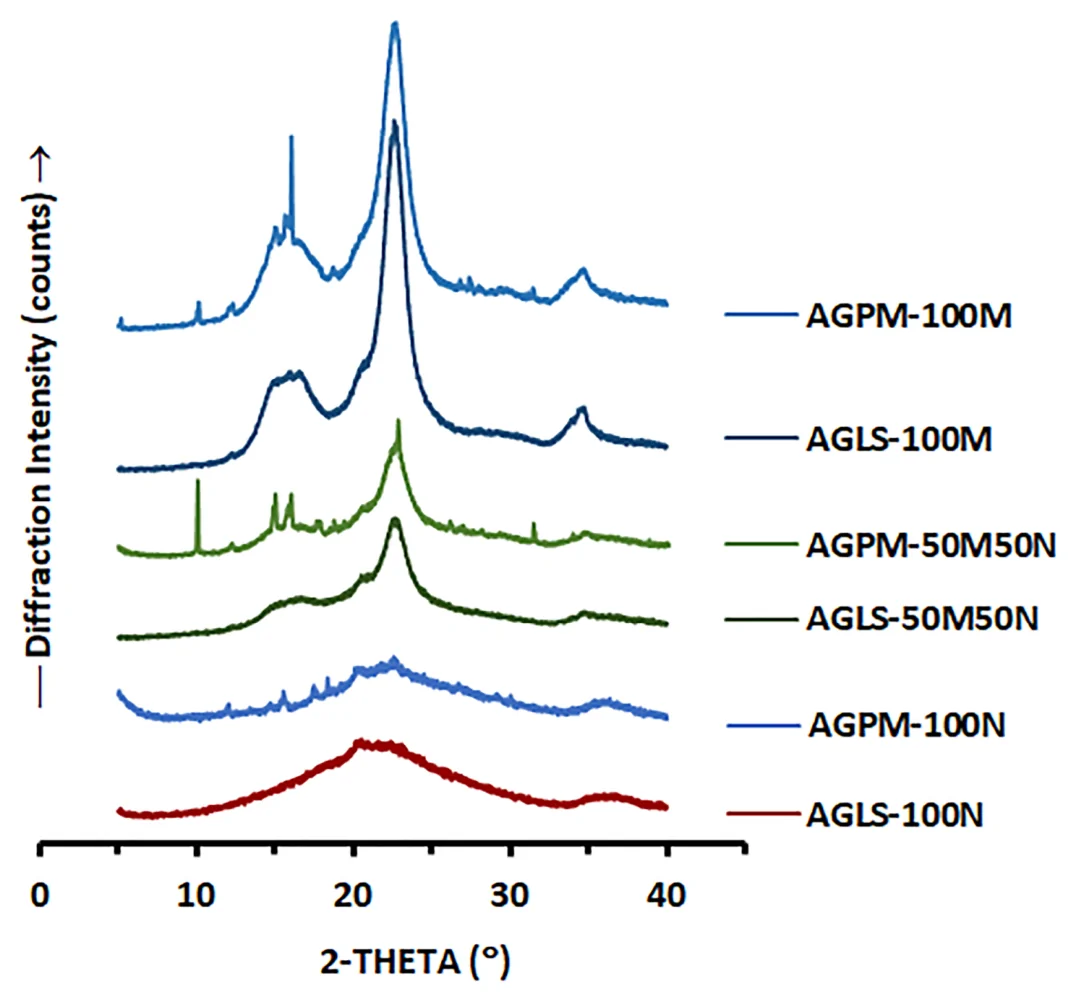
PXRD patterns of AGLSs (AGLS-100M, AGLS-50M50N, and AGLS-100N) in comparison with AGPMs (AGPM-100M, AGPM-50M50N, and AGPM-100N).

PXRD is an effective and significant instrument for assessing the solid-state properties of substances and is a reliable instrument for comparative analysis. The PXRD patterns of AGPM, as shown in Figure 4, reveal that the distinctive peaks of AG were mostly preserved at their initial diffraction angles, in addition to the PXRD patterns of the carriers. AGPM-100N exhibited sharp peaks at 12.0°, 15.5°, 17.5°, 18.3°, 26.7°, 29.1°, and 30.0° 2θ. AGPM-50M50N exhibited intense peaks at 10.0°, 15.0°, 16.0°, 17.6°, 26.1°, 28.1°, and 31.4° 2θ. For AGPM-100M, intense peaks were observed at 10.1°, 12.3°, 15.0°, 16.0°, 26.8°, 27.4°, and 31.5° 2θ. These prominent diffraction peaks were nearly preserved at the original diffraction angles of the AG powder. Prior research has demonstrated that AG displays a prominent crystalline structure, characterized by pronounced, sharp, and significantly dispersed peaks at 2 theta angles between 9.8° and 31.4° [6,7,79,80]. The PXRD pattern of AGLS exhibited significant changes from neat AG powder. Notably, only the characteristic pattern of the carriers was observed, whereas the AG diffraction peak disappeared. It is acknowledged that the absence of PXRD peaks is associated with molecular dispersion or a non-crystalline state. However, this may also arise from dilution or a reduction in API crystallinity falling below the detection threshold, and/or peak obscuration caused by excipients. This situation may arise in the case of AGLS-100M, where the AG loading was recorded at only 1.67%. Nevertheless, considering the FM value of the formulated AGLS, where C_AG_ was lower than S_AG_, the absence of AG diffraction peaks in AGLS likely involves the complete dissolution of AG in 1:2 KHS:NMP and the transition of crystalline AG to a non-crystalline state. Additional evaluations are required to explore and verify the non-crystalline or molecular dispersion state of AG, including an analysis of its thermal properties using differential scanning calorimetry and a quantitative assessment of the PXRD detection limit. Notably, the complete transformation of the API into a non-crystalline state in the liquisolid system can be ascertained when the liquid vehicle has sufficient solvency capacity for the API in the liquid medication [6,7,23,67,72].

It is well recognized that a molecularly dispersed state is crucial for the successful performance of a liquisolid system. The recrystallization or development of AG crystallites after storage under varying circumstances has not been examined in this study. The alterations in the solid state of the AGLS formulation after storage require further evaluation. Nevertheless, considering the S_AG_ and C_AG_ values of all AGLS formulations (with S_AG_ < C_AG_ values and FM = 1), this may assist in preserving the non-crystalline state of AG in the AGLS throughout storage. Previous research has demonstrated that the recrystallization of *A. paniculata* extract in liquisolid powders after three months of storage occurs specifically when the FM parameter is significantly less than 1, leading to the decline of its non-crystalline structure during the evaluated storage duration [6,51].

#### 3.2.4. AG Release Profiles and Parameters

The release of AG from AGLSs was assessed in comparison to AGPMs under both SGF and SIF conditions. Prior research has shown that AG is poorly soluble in SGF, with a solubility value of 0.05 mg/mL (37 °C) [51]. In SIF, a comparable saturated concentration of AG dissolved was noted (0.05 ± 0.01 mg/mL (*n* = 3), 37 °C). Regarding the solubility value (S_AG_), the dosage of AG in the release container (D_AG_), and the release medium volume (V, 100 mL), the computed sink index (SI) (SI = S_AG_/(D_AG_/V)) was found to be 1.0. This suggests that the AG release under the experimental setting in this study occurred under intermediate non-sink conditions. Significantly, the SI calculation in this study omitted the formulation-derived solubilization (NMP and KHS from AGLS), and the C_s_ was specifically confined to the solubility of unadulterated crystalline AG in the SGF (0.1 M HCl) or SIF [81,82,83]. Nevertheless, the AGLS formulation included KHS and NMP, which could influence AG solubility through micellar solubilization and cosolvency, respectively. Consequently, the SI value of the system may exceed 1. Additional investigation into the solubility determination of AG in SGF and SIF with the formulation-equivalent KHS/NMP load shall be conducted to ascertain the actual SI parameter.

Notably, the intermediate non-sink conditions were selected for their potential to identify distinct release behaviors among diverse formulations. The non-sink conditions are crucial for assessing the actual efficacy of supersaturating preparations [81,82,83]. Earlier investigations on drug delivery formulations for AG have concentrated on the release of AG under intermediate non-sink conditions with SI < 3 [6,7,79,84]. A previous report has indicated that the release of AG from liquisolid powder under both intermediate non-sink and sink conditions is comparable [7,51]. The release assessment of AG liquisolid systems based on NMP, polyethylene glycol 6000, and Soluplus as a vehicle, along with porous starch as a carrier, indicated that non-sink conditions distinctly differentiated the release behaviors between the liquisolid formulation, commercial product, and raw material more effectively than sink conditions (medium with 1% SDS), despite the AG release profiles appearing similar under both non-sink and sink conditions [7]. Additionally, the intermediate non-sink medium facilitates simpler media preparation, enhances handling and analysis, and reduces chemical consumption and waste disposal costs.

**Figure 5:**
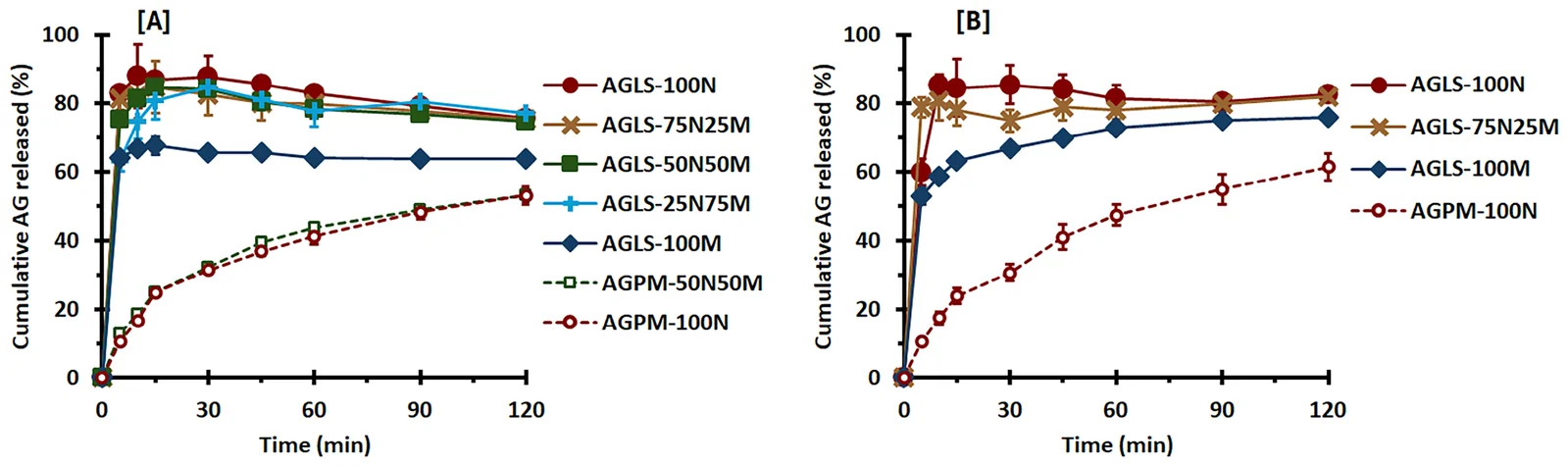
Release patterns of AG from AGLS and AGPM powders in SGF (**A**) and SIF (**B**). Results are presented as mean with SD (*n* = 4).

As shown in Figure 5, AGLSs produced higher release rates and quantities than the AGPMs in both release media. A rapid initial release within 5 min was noted for all AGLS formulations, but a steady delayed release was detected with AGPM. A similar pattern of AG release in SIF to that in SGF was noted for all AGLS and AGPM tested, as their similarity factor (f_2_) was greater than 50. In the context of AGPM’s release in SGF, the Q_30min_ ranged from 31.4% to 32.4% (*p* > 0.05), whereas the RE varied between 36.1% and 39.5% (*p* > 0.05), regardless of the type of carrier used (Table 3). Conversely, AGLS produced 65.6–87.9% of Q_30min_ and 63.9–81.6% of RE in SGF, representing a 2.1–2.8-fold and 1.8–2.3-fold enhancement relative to AGPM-100N. AGLS including NEU as the carrier, especially with a 100% NEU formulation, demonstrated a significantly enhanced AG release (*p* < 0.05).

It is important to note that there was a gradual decrease in the AG release percentage after a swift initial increase, which peaked at approximately 15–30 min. This post-peak decline behavior was clearly observed with the NEU-based AGLS, where the initial surges within 10 min reached 81–88% in SGF and 82–94% in SIF. This resulted in the AG solubility limit in SGF and SIF (0.05 mg/mL) being approached, which renders precipitation from a supersaturated state likely to occur.

The comparable AG release in the SGF and SIF for both AGLS and AGPM powders suggests that the medium pH has an insignificant influence on its release from the powder. The insignificant influence of medium pH aligns with earlier research, which indicates similar release patterns of AG from pure AG, commercial dropping tablets, physical mixtures, and AG-cyclodextrin ternary formulations in 0.1 M HCl, pH 6.8 PBS, and water [85]. The limited release of AG from AGPM has been attributed to the limited solubility of AG in aqueous medium. The improvement in AG release enhancement from the liquisolid system over the physical mixture was consistent with a previous report, which investigated both non-sink and sink conditions (medium with solubilizer, 0.5% sodium dodecyl sulfate) [6,51]. A recent study revealed comparable AG release from liquisolid systems based on neat NMP of crude *A. paniculata* extract and pure AG under non-sink (0.1 M HCl) and sink (0.5% SDS containing 0.1 M HCl) conditions [51].

It is widely reported that the release enhancement mechanisms proposed by liquisolid technology involve a combination of mechanisms. These factors include an increase in the release surface area through deposition on carrier-coating materials, conversion into a non-crystalline or molecularly dispersed state, and enhancement of wettability and localized co-solvent effect facilitated by non-volatile liquid vehicles [1,12,67]. In AGLS, AG was in a dissolved state in the 1:2 KHS:NMP liquid distributed on the surfaces of carrier-coated particles, along with 1:2 KHS:NMP. Consequently, compared to AGPM, the surface area of AG available for release was significantly increased, leading to a higher release performance. The dissolved state of AG (C_AG_ < S_AG_, FM = 1) could induce a prompt release to the release medium. However, because all AGLS developed in this study had the same AG concentration in the 1:2 KHS:NMP, each AGLS contained an identical loading quantity of 1:2 KHS:NMP per AG test dose. Consequently, the varying AG release behaviors observed among different AGLS can be attributed to the differences in the surface area of the AGLS available for the release medium. The lower AG release from MCC-based AGLS compared with those of NEU-based or NEU-containing AGLS is attributable to the greater available surface area of NEU as compared to MCC. Despite the neat MCC-based system (AGLS-100M) exhibiting a larger quantity of solid particles relative to the specified AG quantity, such as 5 mg AG, by nearly three times compared to the NEU-based system (AGLS-100N), the NEU system may still exhibit a significantly greater SSA than that of MCC. As previously stated, NEU exhibited an SSA of 300 m^2^/g, which is approximately 254 times greater than that of MCC, recorded at 1.18 m^2^/g. This leads to a greater available surface area for the AG molecule for the release medium of NEU-based and NEU-containing systems, thereby enhancing the AG release performance. The increased API release in relation to the SSA carrier aligns with findings from prior research [32]. Significantly, the empirical data on the SSA, as well as the particle size and wettability of the carrier-coating systems following loading with the AG-KHS:NMP liquid solution, were not provided in this study. To elucidate the mechanism of AG release from the produced AGLS, additional studies on the SSA, particle size, and wettability of the MCC:NEU systems, in comparison to the system prepared with neat NEU and neat MCC before and after loading with AG-NMP-KHS liquid solution, are suggested.

The incomplete release of AG from AGLS may be attributed to the incomplete desorption of AG from the solid particle. In the case of NEU, its characteristics, which include a high-surface-area porous network, may lead to the entrapment of the AG-1:2 KHS:NMP solution through tight capillary retention, resulting in incomplete release [33,37,86]. Additionally, the non-sink test conditions may also be associated with incomplete release characteristics. It is important to highlight that the incomplete release of AG from the MCC-based liquisolid system of pure AG material and crude *A. paniculata* extract prepared with NMP as a neat liquid vehicle occurred even under sink conditions [51].

#### 3.2.5. Tablettability and Disintegration Ability

Liquisolid powder is a multicomponent mixture that includes an API (AG), a liquid vehicle (1:2 KHS:NMP), and excipient powders (carrier-coating materials). Each component plays a vital role in enhancing the overall strength of the tablet. To assess the capacity of an AGLS to be converted into a tablet with defined mechanical strength under low compression pressure, an analysis of tabletability relationships, specifically the correlation between tablet tensile strength and compaction pressure, was conducted. The related tablet thickness and tensile strength values of all AGLS and AGPM compacts are listed in Table 4. The resulting strength–pressure, or tabletability, profiles of AGLS as compared with AGPM-50M50N are shown in Figure S4A (Supplementary Materials). In general, high compression pressures resulted in dense compacts with reduced thickness. Notably, the thickness of the AGLS (AGLS-50M50N) was significantly reduced compared to that of the corresponding AGPM (AGPM-50M50N). The tensile strength of the AGPM-50M50N compacts exhibited a roughly linear increase with increasing applied pressure across the entire range of compression pressures examined (1.25, 2.5, and 5 MPa). In contrast, for all AGLSs, irrespective of the carrier type, an initial linear strength–pressure relationship with a plateau at a compression pressure above 2.5 MPa was observed. Across all pressures analyzed, the tensile strength of AGLS-100N compacts consistently exceeded that of AGLS-100M. Consequently, the MCC-NEU-based AGLS exhibited superior tensile strength compared to the MCC-based AGLS.

The disintegration ability in SGF (0.1 M HCl) was determined exclusively using compacts compressed at 1.25 MPa. As presented in Table 4 and Figure S4B (Supplementary Materials), the results clearly demonstrate the influence of the carrier type and fabrication on the liquisolid systems. Compared to AGPM-50M50N, the fabrication of AGLS-50M50N resulted in an extended disintegration time. While AGLS-100M disintegrated within a minute, AGLS-100N required up to 89.30 min to disintegrate. Consequently, a higher concentration of NEU within the AGLS correlated with an increased duration of disintegration. The prompt disintegration of AGLS-100M and AGPM-50M50N (1.08–1.16 min) may be relevant to their tensile strength (0.97–1.04 MPa). Furthermore, MCC functions as a disintegrant mainly by wicking or capillary action. The poor mechanical integrity of these compacts facilitates the swift ingress of an aqueous liquid into the porous channels through capillary action, attributed to the hydrophilic and hygroscopic characteristics of MCC. This occurrence would lead to an increase in pore pressure, expansion of pores, disruption of hydrogen bonds between neighboring particles, and eventual disintegration of the compact [87,88]. NEU could enhance disintegration by increasing capillary forces due to its highly porous, amorphous composition. Nonetheless, AGLS containing NEU exhibited considerable prolongation of disintegration, and the effects of prolongation appear to be contingent upon the NEU concentration. This suggests that the coexistence of NEU and MCC in AGLS compacts significantly hinders the disintegration capacity of MCC. For AGLS compacts with NEU at ≥50% (AGLS-50M50N, AGLS-25M75N, and AGLS-100N), they lose the capability to perform as immediate-release tablets, as their disintegration time exceeds 15 min [55].

The prolonged disintegration of these NEU-containing AGLS may be attributable to their greater tensile strength. The densely interconnected AGLS matrix formed by a substantial bonding area and robust interparticulate adhesion during the compaction process may be excessive. Furthermore, it is hypothesized that the 1:2 KHS:NMP located within the porous channels of the compact microstructure forms a liquid bridge, creating capillary and surface tension forces [89] that may restrict the penetrable internal structure of the SGF medium. This restricts the SGF medium’s ability to diffuse effectively across the entire compact, hence constraining the capillary force for overcoming the interparticulate bonding inside the NEU-containing AGLS matrix. In general, the prolongation of tablet disintegration has adversely impacted subsequent release. The incorporation of superdisintegrants such as sodium starch glycolate or croscarmellose sodium is essential for the NEU-containing AGLS mixture, as they facilitate the disintegration of the matrix and promote the expulsion of the contents from the pores [33].

### 3.3. AGLS Tablet Characteristics

#### 3.3.1. Appearance and Tablet Properties

AGLS and AGPM were subsequently formulated into tablets with a dosage of 15 mg of AG, a superdisintegrant (sodium starch glycolate), and a lubricant (magnesium stearate) using a hydraulic press without any supplementary processes. The direct compression approach avoids the issues associated with wet granulation and reduces equipment utilization. Notably, sodium starch glycolate and magnesium stearate are commonly used components in tablet formulations, including liquisolid tablets [30,90,91,92,93,94,95]. The typical amount of sodium starch glycolate employed as a superdisintegrant in tablet formulations varies between 2% and 8%, with an ideal concentration around 4%, but 2% can occasionally suffice. Magnesium stearate is primarily employed as a lubricant in the manufacturing of capsules and tablets, often at concentrations between 0.25% and 5.0% *w*/*w* [30]. In this study, constant proportions of SSG and magnesium stearate were utilized at 4% and 1%, respectively. Given that excessive hardness of the tablet can impact its disintegration capability, the AGLSTs and AGPMTs were designed to achieve a hardness of 60 ± 10 N.

All AGLST and AGPMT tablets were successfully fabricated. The resulting tablets had smooth surfaces with a uniform appearance, were free of stains, and maintained a consistent shape (Figure 2). The tablet characterization results presented in Table 5 show small and acceptable weight variations according to the pharmacopeial limit [55]. The variation in tablet weight (351.7–990.3 mg) and thickness (2.74–7.12 mm) was related to the loading capacity of each AGLST formulation. The greater thickness of AGPMT-100N compared to that of AGPMT-50M50N can be attributed to the greater bulk density of AGPM-100N. The tablets exhibited average hardness values ranging from 52.0 to 69.4 N and a friability of less than 1%, indicating that all formulations achieved acceptable mechanical properties. The difference in tablet hardness between the formulations was related to their tabletability. Both AGPMT and AGLST-100M disintegrated within 0.14–0.15 min. For NEU-containing AGLST, a disintegration time of 5.18–15.03 min was observed. The significant prolongation of the disintegration of NEU-containing AGLST was related to the disintegration ability and mechanical strength of the NEU-based AGLS compacts. The better disintegrability of AGLST compared with that of the AGLS compact was due to the effect of sodium starch glycolate, which functions as a superdisintegrant.

Significantly, all AGLSTs were fabricated at slightly different compression pressures to obtain tablets with the desired hardness. For AGLST-100N, AGLST-75N25M, AGLST-50N50M, and AGLST-25N75M, a compression load of 1.25 MPa and a 30 s holding time were used. For AGLST-100M, AGPMT-100N, and AGPMT-50N50M, a compression load of 1.0 MPa and a 30 s holding time were used. Different AGLSTs exhibited notable variations in dosage unit size (351.7–990.3 mg per tablet) owing to differences in AG loading and the use of slightly varying compression pressures. This may affect the porosities of the final tablets. The large variation in the total volume of the AGLS powder can influence the stress distribution in the die cavity during compression, thereby changing the residual intraparticle and interparticle porosities of the AGLSTs. Such porosity configuration is often a determinant of capillary action and the amount of water infiltration, which, in turn, affects tablet disintegration and AG release. The differences in the disintegration times of the different AGLSTs may also arise from the differences in their intraparticle and interparticle porosities. The lower relative compaction density of AGLST-100M (~990 mg) may have resulted in a highly porous microstructure. This bulky arrangement enhanced the capillary action of water for fast water imbibition and rapid disintegration. For AGLST-100N (~352 mg), although a slight compression pressure was used, the 2.8-fold decrement in the powder size may have caused a denser structure with low porosity, and thus slower disintegration.

#### 3.3.2. AG Content and Release Behaviors

The AG content in the formulated AGLST was 14.8–15.2 mg, corresponding to 95.4–98.7% of the theoretical loading (Table 6). The AG content, with a low SD of less than 2.3%, indicates a consistent AG content in tablets. For AGPMT, the determined AG loading percentage was within the acceptable range.

**Figure 6:**
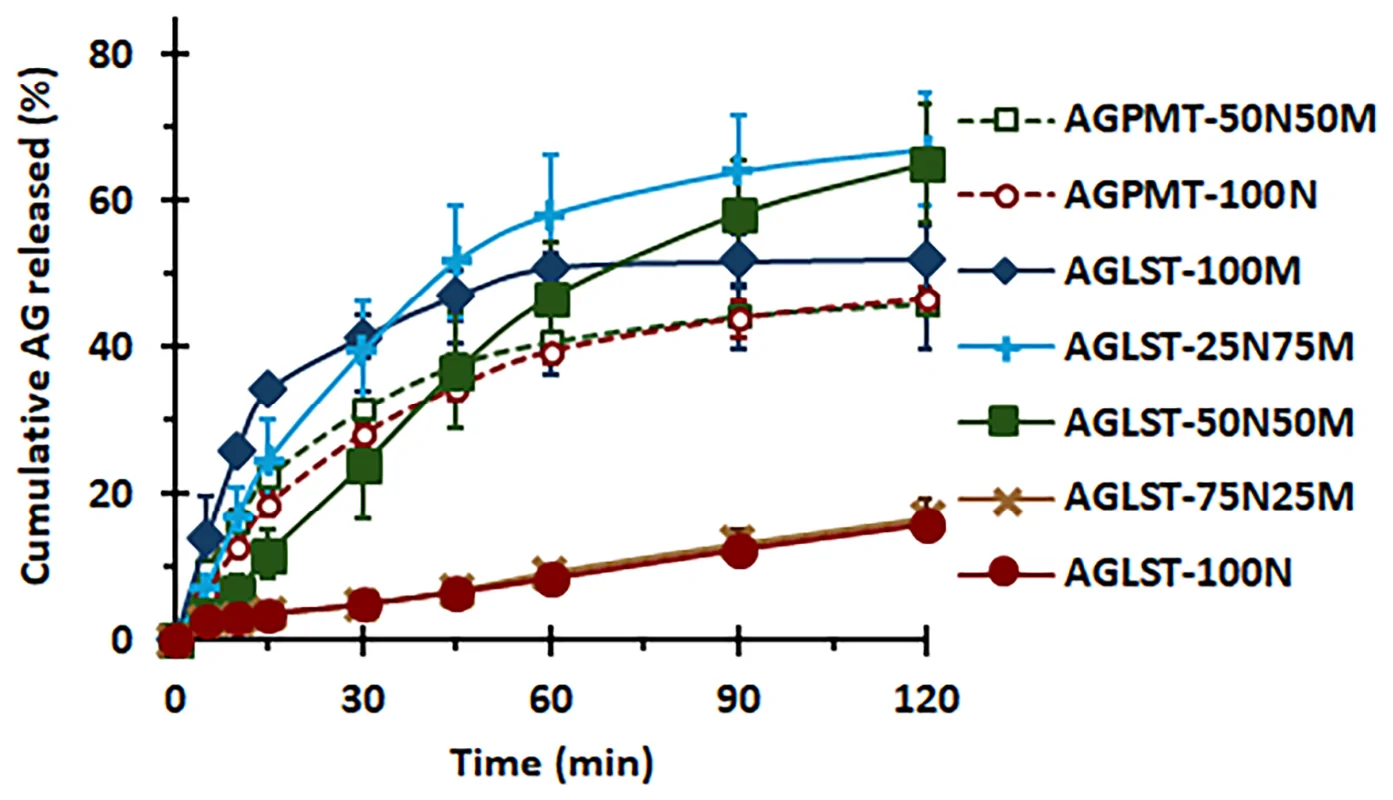
In vitro release pattern of AG from AGLSTs and AGPMTs in SGF. Results are presented as mean ± SD (*n* = 6).

The AG release from the AGLSTs and AGPMTs was performed using the paddle method. It should be noted that the USP paddle method, also referred to as USP Apparatus 2, is the standard and most commonly utilized in vitro release technique for assessing the release of APIs or active phytochemicals from liquisolid systems [7,25,34,90,91,94,95,96,97]. According to the standard guidelines [55], the agitation speed of apparatus 2 (paddles) should be set at either 50 or 75 rpm for immediate-release oral formulations. In this study, a mild agitation condition of 50 rpm was employed to preserve the discriminatory power. Increased shear forces can lead to an accelerated release by eroding or breaking down the dosage form more quickly than it would occur in vivo, which may produce inaccurately favorable API release profiles. The SI for the release test of the AGLSTs and AGPMTs was 2.0, considering intermediate non-sink conditions. Significantly, the SI calculation in this study omits the formulation-derived solubilization (NMP and KHS from AGLST), and the C_s_ is specifically confined to the solubility of unadulterated crystalline AG in the SGF (0.1 M HCl) [53,54,55]. The release profiles of AG from AGLST and AGPMT in the SGF are illustrated in Figure 6, accompanied by the release parameters detailed in Table 6. In every instance, the AG was gradually released from the tablet, with Q_30min_ values varying between 5.0 ± 0.3% and 41.4 ± 3.0% for AGLSTs, and from 28.1 ± 1.3% to 31.5 ± 2.6% for AGPMTs. Overall, the AG release from the tablet form was inferior to that of the powder form. While the AG release from both AGPMTs was comparable, that from AGLSTs varied significantly. AGLST-100M exhibited the highest performance in AG release, showcasing the best Q_30min_ and RE values. The greatest AG release from AGLST-100M may arise from the bulky arrangement with a more porous microstructure, which facilitates greater water imbibition, thus promoting disintegration and AG release. For the AGLSTs with the MCC:NEU mixture, the release of AG was observed to be more delayed with an increased concentration of NEU. For AGLST-100N and AGLST-25M75N, their Q_30min_ values were only 5.0%, while the RE after 120 min was 8.7–9.2%.

The release of AG from various AGLSTs was related to the disintegration ability of the tablets. The AG molecules emerged and dissolved in the release medium following tablet disintegration. For AGLST-100M, fast and complete disintegration occurred within a few minutes upon contact with the release medium. However, incomplete release may be related to the incomplete desorption of AG from the solid particles of the AGLST. Moreover, the compression procedure during tableting directly alters the pore network and structure of MCC, which may dictate the available surface area of the AG exposed to the release medium.

For NEU-containing AGLSTs, observations found incomplete disintegration of the NEU-containing AGLSTs. After 2 h of investigation, a large fraction of AGLST-100N and AGLST-25M75N remained predominantly intact at the bottom of the release vessel, exhibiting only minor disintegration into large aggregates instead of forming discrete free particles. Generally, disintegration of the tablet in a release vessel occurs mainly due to the wicking effect or the imbibition of aqueous medium into the tablet matrix [89]. The inability of NEU to swell in water [30] resulted in the mild and limited disintegration of AGLST-100N and AGLST-25M75N under gentle hydrodynamic conditions caused by paddle rotation, despite the use of swelling agents such as sodium starch glycolate as a superdisintegrant. Moreover, the compression process also resulted in the secure entrapment of AG-1:2 KHS:NMP within the compact and dense mesoporous internal matrix of NEU. This, together with the inability of NEU to swell and dissolve in water [30], significantly influenced the accessible surface area for the liberation of AG. This aligns with an earlier report indicating that the release percentage of rosuvastatin was only approximately 11–25% within 30 min, attributed to the lower disintegration of NEU-based liquisolid tablets [29]. Incomplete API release from compressed compacts of NEU with adsorbed liquid and lipid-based formulations is a recognized issue [3,33,37,38,39,40,41]. Van Speybroeck et al. [37] reported that incomplete desorption of the liquid excipients of self-emulsifying drug delivery systems from the inorganic mesoporous carrier, such as NEU, reduces their in vitro release and thus in vivo bioavailability performance. In fact, release retardation also occurs with conventional tablets containing NEU. A previous study reported a significant decrease in the release rate and quantity of resveratrol from tablets with a NEU content of 10–30% *w*/*w* [98]. In contrast, the adequate mechanical strength combined with the poor breakdown characteristics of NEU-based liquisolid tablets may provide extended API release through the liquisolid system [3]. Kanojiya et al. [38] utilized the sustained-release properties of NEU-based liquisolid to develop a liquisolid compact-based vaginal tablet containing an antifungal API.

To establish a clear correlation between the internal geometry of the AGLSTs and their release profiles, the porosity of the AGLSTs shall be further determined. Tablet porosity is a crucial parameter that directly influences liquid medium transport, swelling, matrix disintegration, and subsequent release of the API. Additionally, it is essential to note that the findings of this study were derived from technical replicates (individual tablets sampled from a single representative batch of each formulation) to evaluate the within-batch pharmaceutical quality and uniformity. The reproducibility of the release performance and enhancements across independent preparation batches shall be further assessed.

It is important to acknowledge that this study primarily focused on analyzing the physicochemical properties and release behavior of the model phytochemical AG from several liquisolid systems created with MCC, NEU, and NEU:MCC combinations as carriers. Current AG liquisolid formulations have not been directly developed based on existing residual solvent guidelines. KHS:NMP at a 1:2 ratio served as a model liquid vehicle that fulfilled the solvation requirements of AG in the developed liquisolid systems. It is acknowledged that the choice of solvent must take into account pharmaceutical acceptability, compatibility, safety, and the designated method of administration. According to the AGLST formulation composition (Table 1), administering a dose comparable to 15 mg of AG would theoretically yield KHS and NMP exposures of approximately 45 mg and 90 mg, respectively. The estimated NMP exposure after AGLST ingestion significantly surpassed the existing daily allowable exposure threshold for NMP. In the context of developing liquisolid formulations for AG compound, subsequent research should focus on identifying an orally acceptable solvent system that meets the solvation criteria for AG while complying with regulatory safety standards. Such systems can be constructed utilizing ternary co-solvent combinations, pharmaceutically acceptable deep eutectic mixtures, and hydrotrope-cosolvent amalgams.

## 4. Conclusions

This study comparatively demonstrated the physicochemical properties and release characteristics of andrographolide liquisolid systems developed using microcrystalline cellulose, magnesium aluminometasilicate, and their combinations as carriers. Five different andrographolide liquisolid powders and tablets with varying microcrystalline cellulose and magnesium aluminometasilicate ratios were prepared. This study demonstrates for the first time that the flowable liquid retention potential in a binary carrier mixture may be anticipated based on the weighted contributions and flowable liquid retention potential of its constituent components. Blending 25–75% of magnesium aluminometasilicate with microcrystalline cellulose significantly improved liquid medication loading and, thus, the andrographolide loading by 1.7–2.6 times. Consequently, the tablet size was effectively reduced by 1.6–2.8 times. The andrographolide release efficiency from the liquisolid powder in both simulated gastric fluid and intestinal fluid significantly increased with the blends of microcrystalline cellulose and magnesium aluminometasilicate by about 2 times compared with the physical mixture. The presence of magnesium aluminometasilicate improved the tabletability of microcrystalline cellulose-based compacts, with greater tensile strength and thus retarded disintegration. Nevertheless, for liquisolid tablets based on magnesium aluminometasilicate-rich carriers, investigation and optimization of the optimal superdisintegrant and tablet components are required to facilitate swift disintegration and consequently sustain the enhanced andrographolide release of the liquisolid powder formulations.

The current study effectively demonstrates the potential of liquisolid formulations based on microcrystalline cellulose and magnesium aluminometasilicate blends to improve the loading and release properties of andrographolide; however, they were not specifically developed or optimized in accordance with established regulatory safety criteria, including the ICH Q3C guidelines for residual solvents. In the context of developing liquisolid formulations for andrographolide, further investigations must prioritize the discovery of an orally acceptable solvent system that fulfills the solvation requirements for AG and adheres to regulatory safety protocols.

## Figures and Tables

**Table 1:** Composition of AGLSs and AGPMs.

AG Formulations	Carriers	Liquid Load Factor	Composition Quantity (g)			
			AG	1:2 KHS:NMP	Carriers	Aerosil
AGLS-100M	MCC	0.202	1.61	14.52	79.87	3.99
AGLS-75M25N	75:25 MCC:NEU	0.350	2.67	24.03	69.81	3.49
AGLS-50M50N	50:50 MCC:NEU	0.563	3.48	31.31	62.11	3.11
AGLS-25M75N	25:75 MCC:NEU	0.744	4.14	37.28	55.79	2.79
AGLS-100N	NEU	0.925	4.68	42.15	50.64	2.53
AGPM-100N	NEU	N/A	5.00	0	90.50	4.50
AGPM-50M50N	50:50 MCC:NEU	N/A	5.00	0	90.50	4.50

M represents the MCC, and N denotes NEU. The liquid load factor indicates the highest amount of 1:2 KHS:NMP per gram of carrier that maintains the acceptable flowability of the powder mixture. N/A = Not applicable.

**Table 2:** Bulk density, flowability, and AG loading of AGLSs and AGPMs.

AG Powder Formulations	Bulk Density ^1^ (g/mL)	Flowability ^1^		AG Loading ^2^	
		Angle of Slide (°)	Carr Index (%)	Actual Loading Percentage (% *w*/*w*)	Percent Label Amount (% *w*/*w*) *
AGLS-100M	0.41 ± 0.01	22.7 ± 2.3	11.6 ± 1.1	1.63 ± 0.03	101.16 ± 1.56
AGLS-75M25N	0.35 ± 0.01	21.3 ± 3.8	14.1 ± 1.7	2.72 ± 0.01	101.72 ± 0.23
AGLS-50M50N	0.33 ± 0.01	21.0 ± 2.0	11.0 ± 1.8	3.55 ± 0.03	101.86 ± 0.99
AGLS-25M75N	0.30 ± 0.01	21.3 ± 3.1	15.7 ± 0.9	4.20 ± 0.02	101.19 ± 0.57
AGLS-100N	0.28 ± 0.01	23.7 ± 2.1	16.6 ± 0.1	4.64 ± 0.04	99.11 ± 0.84
AGPM-100N	0.15 ± 0.01	20.3 ± 1.5	20.5 ± 0.5	4.88 ± 0.45	101.08 ± 6.95
AGPM-50M50N	0.19 ± 0.01	20.7 ± 1.2	18.5 ± 0.3	4.90 ± 0.27	99.59 ± 3.72

Results are presented as mean with SD of ^1^ *n* = 3 and ^2^ *n* = 4. * The percent label amount indicates the actual quantity of AG present in a liquisolid powder relative to the theoretical quantity that was originally incorporated during manufacturing.

**Table 3:** Release parameters of AGLSs and AGPMs in SGF and SIF.

AG Powder Formulations	SGF		SIF		f_2_
	Q_30min_ (%)	RE (%)	Q_30min_ (%)	RE (%)	
AGLS-100M	65.6 ± 0.8 ^a^	63.9 ± 1.6 ^a^	66.0 ± 1.2 ^a^	67.9 ± 2.4 ^a^	55
AGLS-75M25N	84.7 ± 0.5 ^b^	77.6 ± 1.1 ^b^	ND	ND	ND
AGLS-50M50N	84.1 ± 1.6 ^b^	77.4 ± 1.0 ^b^	ND	ND	ND
AGLS-25M75N	82.7 ± 6.3 ^b^	77.8 ± 2.4 ^b^	76.9 ± 3.2 ^b^	79.4 ± 2.3 ^b^	69
AGLS-100N	87.9 ± 6.1 ^b^	81.6 ± 2.0 ^b^	90.9 ± 6.3 ^c^	79.5 ± 2.9 ^b^	53
AGPM-100N	31.4 ± 1.1 ^c^	36.1 ± 3.5 ^c^	31.1 ± 2.4 ^d^	43.0 ± 3.2 ^c^	65
AGPM-50M50N	32.4 ± 0.8 ^c^	39.5 ± 0.9 ^c^	ND	ND	ND

Results are presented as mean ± SD (*n* = 4). ND = not determined. ^a–d^ Means in the identical column with the same superscript letter are regarded as similar (*p* > 0.05). Q_30min_ (%) denotes the total release amount of AG at the 30 min mark. RE denotes the release efficiency at 120 min. f_2_ indicates the similarity factor between the release profile in SGF and SIF.

**Table 4:** Thickness, tensile strength, and disintegration time of compacts fabricated from AGLS and AGPM powders as a function of compression pressure.

AG Formulations	Compression Pressure (MPa)	Thickness (mm) ^1^	Tensile Strength (MPa) ^2^	Disintegration Time (min) ^3^
AGLS-100M	1.25	3.40 ± 0.03 ^a^	1.04 ± 0.04 ^a^	1.16 ± 0.01 ^a^
	2.5	3.15 ± 0.02 ^b^	2.00 ± 0.02 ^b^	ND
	5.0	3.12 ± 0.01 ^b^	2.20 ± 0.09 ^b^	ND
AGLS-75M25N	1.25	3.54 ± 0.03 ^a^	1.34 ± 0.06 ^c^	9.68 ± 0.54 ^b^
	2.5	3.11 ± 0.01 ^b^	2.94 ± 0.09 ^d^	ND
	5.0	3.09 ± 0.01 ^b^	3.24 ± 0.09 ^e^	ND
AGLS-50M50N	1.25	3.54 ± 0.01 ^a^	1.66 ± 0.06 ^c^	48.49 ± 1.05 ^c^
	2.5	3.04 ± 0.01 ^b^	3.05 ± 0.07 ^d^	ND
	5.0	2.90 ± 0.28 ^b^	3.20 ± 0.10 ^e^	ND
AGLS-25M75N	1.25	3.54 ± 0.02 ^a^	1.79 ± 0.10 ^b^	66.24 ± 3.17 ^d^
	2.5	2.93 ± 0.02 ^b^	2.81 ± 0.18 ^d^	ND
	5.0	2.82 ± 0.17 ^b^	3.01 ± 0.23 ^d^	ND
AGLS-100N	1.25	3.50 ± 0.02 ^a^	1.65 ± 0.12 ^c^	89.30 ± 1.45 ^e^
	2.5	2.99 ± 0.02 ^b^	2.84 ± 0.50 ^d^	ND
	5.0	2.90 ±0.03 ^b^	3.24 ± 0.30 ^e^	ND
AGPM-50M50N	1.25	5.09 ± 0.07 ^c^	0.97 ± 0.11 ^a^	1.08 ± 0.01 ^a^
	2.5	3.74 ± 0.07 ^a^	3.51 ± 0.46 ^e^	ND
	5.0	2.82 ± 0.99 ^b^	6.28 ± 0.33 ^f^	ND

Results are presented as mean with SD of ^1^ *n* = 10, ^2^ *n* = 6, and ^3^ *n* = 3. ND = not determined. ^a–f^ Means in the identical column with the same superscript letter are regarded as similar (*p* > 0.05).

**Table 5:** Tablet properties and release parameters of AGLSTs and AGPMTs.

AG Tablet Formulations	Tablet Weight (mg) ^1^	Tablet Thickness (mm) ^1^	Friability (%) ^2^	Hardness (N) ^3^	Disintegration Time (min) ^4^
AGLST-100M	990.3 ± 2.1	7.12 ± 0.04	0.10	54.7 ± 3.3 ^a^	0.27 ± 0.05 ^a^
AGLST-75M25N	608.5 ± 3.0	4.53 ± 0.06	0.29	61.0 ± 5.5 ^b^	5.18 ± 0.21 ^b^
AGLST-50M50N	464.8 ± 1.0	3.64 ± 0.05	0.04	62.8 ± 2.1 ^b^	12.28 ± 0.16 ^c^
AGLST-25M75N	395.3 ± 1.2	3.09 ± 0.04	0.01	69.4 ± 2.2 ^b^	14.64 ± 0.65 ^d^
AGLST-100N	351.7 ± 1.2	2.74 ± 0.03	0.01	68.2 ± 6.2 ^b^	15.03 ± 0.03 ^d^
AGPMT-100N	331.2 ± 1.8	5.29 ± 0.08	0.74	52.0 ± 10.3 ^a^	0.14 ± 0.01 ^a^
AGPMT-50M50N	324.4 ± 1.0	3.55 ± 0.04	0.07	53.7 ± 4.5 ^a^	0.14 ± 0.01 ^a^

Results are presented as mean with SD of ^1^ *n* = 20, ^2^ *n* = 6,^3^ *n* = 4, ^4^ *n* = 3. ^a–d^ Means in the identical column with the same superscript letter are regarded as similar (*p* > 0.05).

**Table 6:** AG content uniformity and release parameters in SGF of AGLSTs and AGPMTs.

AG Tablet Formulations	AG Content Uniformity ^1^		Release Parameters ^2^	
	AG Content (mg/Tablet)	Percent Label Amount (% *w*/*w*)	Q_30min_ (%)	RE (%)
AGLST-100M	15.1 ± 0.1	98.7 ± 0.4	41.4 ± 3.0 ^a^	44.6 ± 2.5 ^a^
AGLST-75M25N	14.8 ± 0.4	95.4 ± 2.3	39.4 ± 7.0 ^a^	49.9 ± 6.7 ^a^
AGLST-50M50N	14.9 ± 0.2	96.8 ± 1.3	23.9 ± 7.1 ^b^	40.4 ± 5.9 ^b^
AGLST-25M75N	15.1 ± 0.5	98.1 ± 2.9	5.0 ± 0.4 ^c^	9.2 ± 1.1 ^c^
AGLST-100N	15.2 ± 0.2	98.1 ± 1.3	5.0 ± 0.3 ^c^	8.7 ± 0.7 ^c^
AGPMT-100N	14.9 ± 0.9	95.7 ± 6.1	28.1 ± 1.3 ^d^	34.0 ± 1.1 ^b^
AGPMT-50M50N	15.1 ± 0.2	98.0 ± 1.5	31.5 ± 2.6 ^d^	36.1 ± 3.5 ^b^

Results are presented as mean with SD of ^1^ *n* = 4, ^2^ *n* = 6. ^a–d^ Means in the identical column with the same superscript letter are regarded as similar (*p* > 0.05). The percent label amount indicates the actual quantity of AG present in a tablet relative to the theoretical quantity that was originally incorporated during manufacturing.

## Data Availability

The original contributions presented in this study are included in the article/Supplementary Material. Further inquiries can be directed to the corresponding author.

## Supplementary Materials

The following supporting information can be downloaded at https://www.mdpi.com/article/10.3390/polym18212667/s1: Table S1: Weighing records of AGLS and AGPM preparation (50 g batch size); Figure S1: Representative HPLC chromatograms of AGLS formulation (AGLS-100N): content [A] and release [B] determination, along with AG standard solution [C]; Figure S2: The relationship between the angle of slide and weight ratio of liquid vehicle (1:2 KHS:NMP) to solid particles (MCC, NEU, and Aerosil). The regression formula correlating the mean values of the angle of slide (*y*-axis) and the corresponding weight ratio of 1:2 KHS:NMP to solid particles (*x*-axis) for MCC, NEU, and Aerosil were y = 37.516x + 26.804 (R^2^ = 0.956), y = 12.510x + 21.890 (R^2^ = 0.943), and y = 8.261x + 26.901 (R^2^ = 0.941), respectively. The Φ_Ca_ values for 1:2 KHS:NMP of MCC and NEU were 0.165 and 0.888, respectively. The Φ_Co_ value for 1:2 KHS:NMP of Aerosil was 0.738; Figure S3: Original images of AGPM and AGPMT as well as AGLSs and AGLSTs: [A] AGPM-100N and AGPMT-100N, [B] AGLS-100N and AGLST-100N, and [C] AGLS-100M and AGLST-100M; Figure S4: [A] Tabletability (mean ± SD, *n* = 6) and [B] disintegration time in 0.1 M HCl (mean ± SD, *n* = 3) of AGLS and AGPM; Table S2: ATR-FTIR band assignments of AG, AG solution in KHS and 1:2 KHS:NMP as well as neat KHS and mixture of 1:2 KHS:NMP, AGLS (AGLS-100N, AGLS-25M75N, AGLS-50M50N, and AGLS-100M), physical mixture of AG with blank liquisolid powder ((AG)(B-LS-100N)-PM and (AG)(B-LS-100M)-PM) along with blank liquisolid powder (B-LS-100N and B-LS-100M).

## Abbreviations

The following abbreviations are used in this manuscript:

Aerosil	Colloidal silicon dioxide
*A. paniculata*	*Andrographis paniculata*
AG	Andrographolide
API	Active pharmaceutical ingredient
ATR	Attenuated total reflection
EtOH	Ethyl alcohol, 99.8%
f_2_	Similarity factor
FTIR	Fourier transform infrared spectroscopy
HCl	Hydrochloric acid
HPLC	High-performance liquid chromatography
LS	Liquisolid
KHS	Polyoxyl 15 hydroxystearate, or Kolliphor HS 15 (KHS)
MCC	Microcrystalline cellulose (MCC)
NEU	Neusilin^®^ US2
NMP	N-Methyl-2-pyrrolidone
PBS	Phosphate-buffered saline
P-gp	P-glycoprotein
PM	Physical mixture
PXRD	Powder X-ray diffraction
Q_30min_	Percentage cumulative release of AG at 30 min
RE	Release efficiency within 120 min
RH	Relative humidity
SD	Standard deviation
SGF	Simulated gastric fluid
SIF	Simulated intestinal fluid
SSA	Specific surface area
