# Supplementary material for: Physicochemical and Release Characteristics of Andrographolide Liquisolid Systems Fabricated Based on Microcrystalline Cellulose, Magnesium Aluminometasilicate, and Their Blends
Source: Polymers (Basel). 2026 Oct 28;18(21):2667. doi: 10.3390/polym18212667 (PMC13766134; doi:10.3390/polym18212667)
Supplement: Supplementary file 1 [file polymers-18-02667-s001.zip › polymers-4621641-supplementary.pdf]

Supplementary Materials

Physicochemical and release characteristics of andrographolide liquisolid systems fabricated based on microcrystalline cellulose, magnesium aluminometasilicate, and their blends

Napaphak Jaipakdee [1,2], Peera Tabboon [1,2], Pinpair Ekkanjanakorn [1], Kattiya Sonchiangkham [1,2], Thaned Pongjanyakul [1], Ekapol Limpongsa[3,*]

[1] Faculty of Pharmaceutical Sciences, Khon Kaen University, Khon Kaen 40002, Thailand; jsaowanee@kku.ac.th (N.J.); peerata@kku.ac.th (P.T.); pinpair.e@kkumail.com (P.E.); kattiya.s@kkumail.com (K.S.); thaned@kku.ac.th (T.P.)

[2] Center for Research and Development of Herbal Health Products, Khon Kaen University, Khon Kaen 40002, Thailand; nj.jaipakdee@gmail.com (N.J.); peerata@kku.ac.th (P.T.)

[3] College of Pharmacy, Rangsit University, Pathum Thani 12000, Thailand; ekapol.l@rsu.ac.th (E.L.)

* Correspondence: ekapol.l@rsu.ac.th; Tel.: +66 8 0519 4956, Fax: +66 2791 6000 ext 1403

Table S1. Weighing records of AGLS and AGPM preparation (50 g batch size).

AG formulations	Carriers	Composition quantity (g)			
		AG	1:2 KHS:NMP	Carriers	Aerosil
AGLS-100M	MCC	0.806	7.255	39.955	2.001
AGLS-75M25N	75:25 MCC:NEU	1.340	12.115	34.910	1.746
AGLS-50M50N	50:50 MCC:NEU	1.750	15.711	31.102	1.650
AGLS-25M75N	25:75 MCC:NEU	2.077	18.661	27.898	1.398
AGLS-100N	NEU	2.335	21.036	25.250	1.250
AGPM-100N	NEU	2.450	0.000	45.910	2.381
AGPM-50M50N	50:50 MCC:NEU	2.475	0.000	45.525	2.298

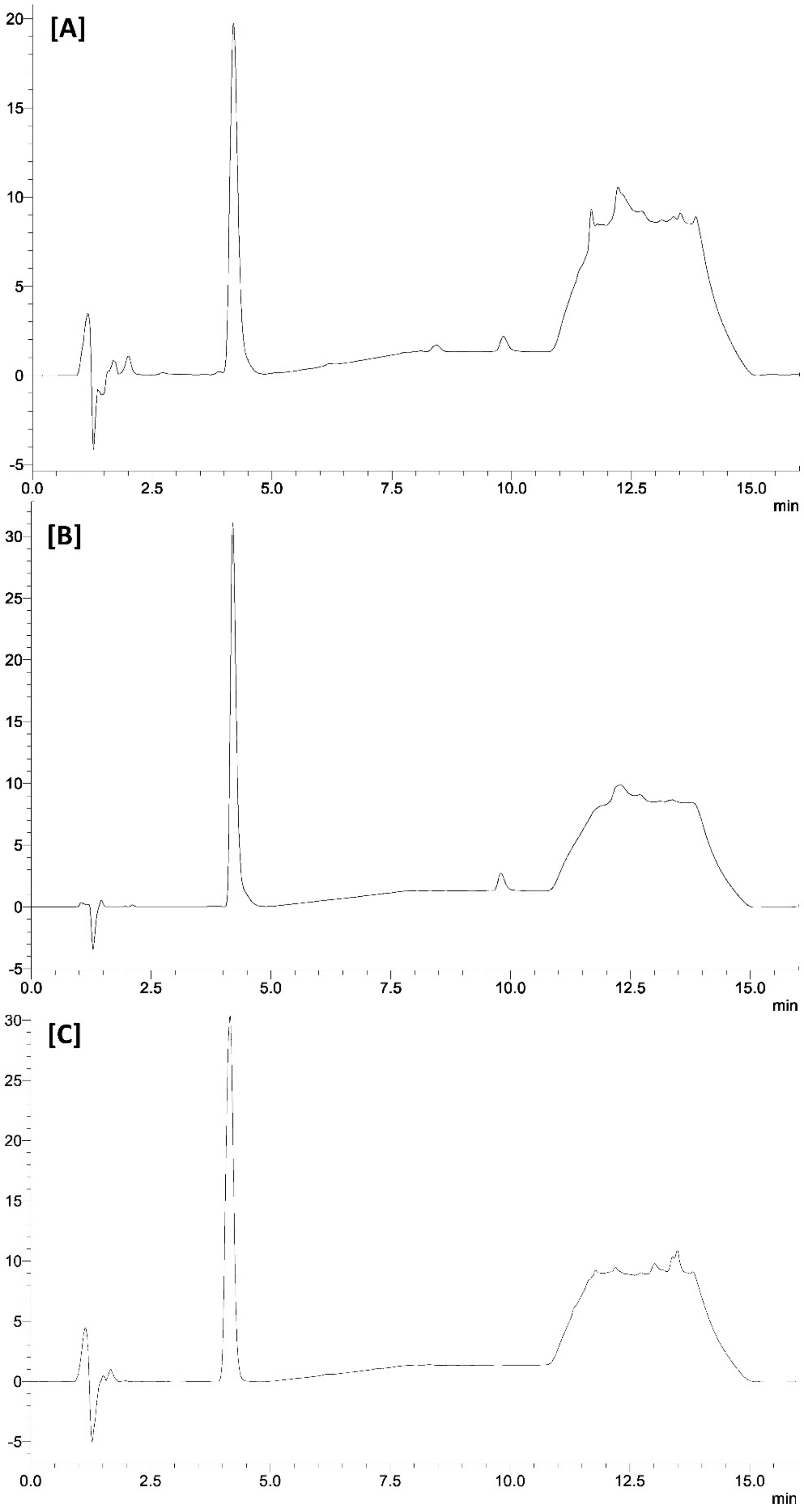

Figure S1. Representative HPLC chromatograms of AGLS formulation (AGLS-100N): content [A] and release [B] determination, along with AG standard solution [C].

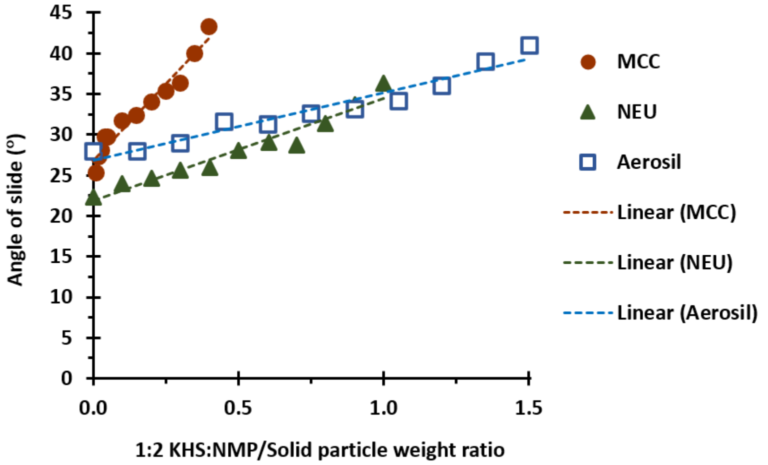

Figure S2. The relationship between the angle of slide and weight ratio of liquid vehicle (1:2 KHS:NMP) to solid particles (MCC, NEU, and Aerosil). The regression formula correlating the mean values of the angle of slide (y-axis) and the corresponding weight ratio of 1:2 KHS:NMP to solid particles (x-axis) for MCC, NEU, and Aerosil were $y = 37.516x + 26.804$ ($R^2 = 0.956$), $y = 12.510x + 21.890$ ($R^2 = 0.943$), and $y = 8.261x + 26.901$ ($R^2 = 0.941$), respectively. The Φ_{Ca} values for 1:2 KHS:NMP of MCC and NEU were 0.165 and 0.888, respectively. The Φ_{Co} value for 1:2 KHS:NMP of Aerosil was 0.738.

[A]

[B]

[C]

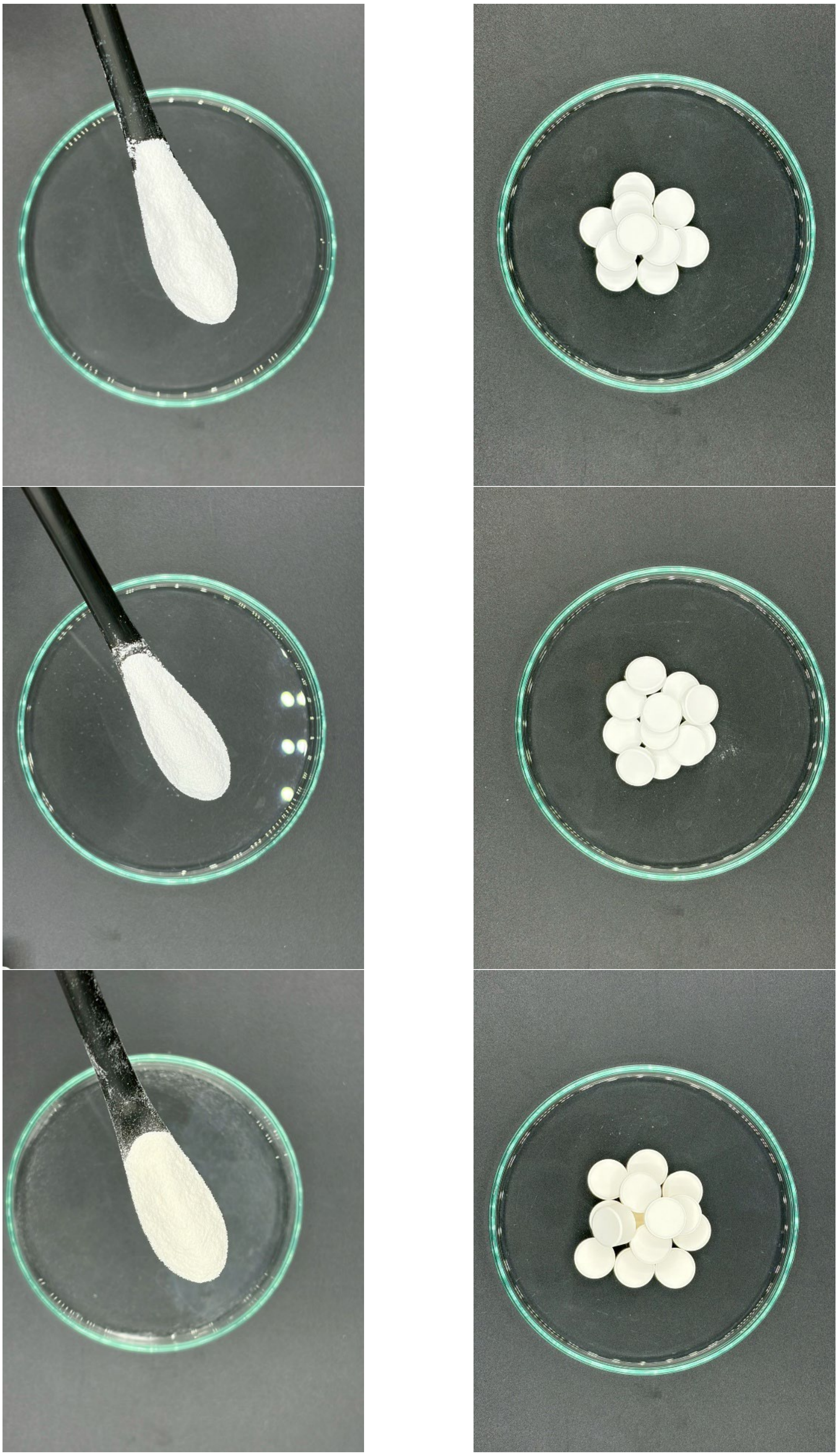

Figure S3. Original images of AGPM and AGPMT as well as AGLSs and AGLSTs: [A] AGPM-100N and AGPMT-100N, [B] AGLS-100N and AGLST-100N, and [C] AGLS-100M and AGLST-100M.

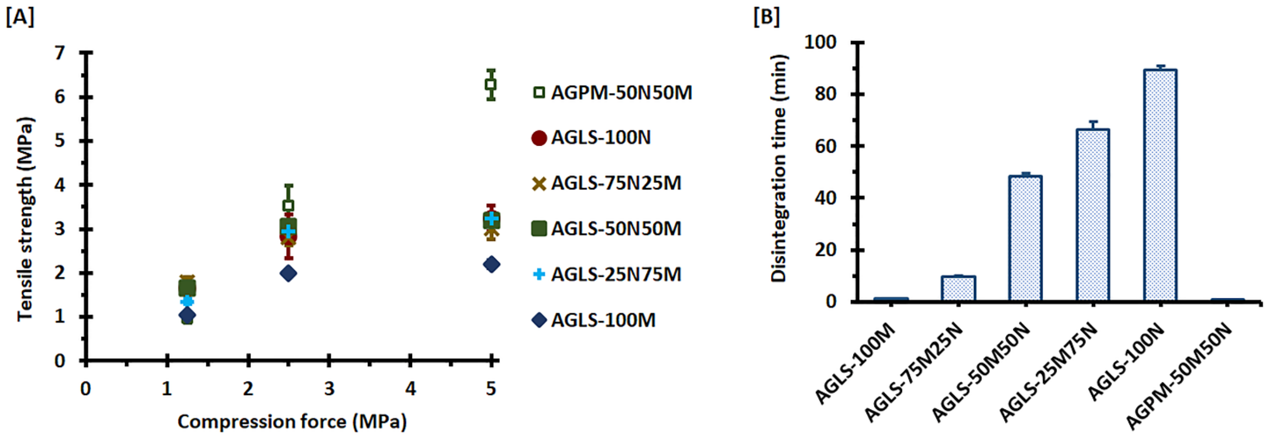

Figure S4. [A] Tabletability (mean ± SD, n = 6) and [B] disintegration time in 0.1 M HCl (mean ± SD, n = 3) of AGLS and AGPM; Table S2. FTIR band assignments of AGLS and AGPM.

Table S2. ATR-FTIR band assignments of AG, AG solution in KHS and 1:2 KHS:NMP as well as neat KHS and mixture of 1:2 KHS:NMP, AGLS (AGLS-100N, AGLS-25M75N, AGLS-50M50N, and AGLS-100M), physical mixture of AG with blank liquisolid powder ((AG)(B-LS-100N)-PM and (AG)(B-LS-100M)-PM) along with blank liquisolid powder (B-LS-100N and B-LS-100M).

AG and formulations	Peak wavenumber (cm^{-1}) and associated functional groups and vibrations								
	O–H stretching	C–H stretching	C=O stretching	C=C stretching	Aromatic ring C=C skeletal vibration	C–H bending	C–O stretching	C–O–C stretching / skeletal vibration	Out-of-plane bending, skeletal peaks
AG	3390, 3289	2980, 2927, 2848	1722	1674		1455	1364, 1294, 1219	1074, 1031	907, 656
10% AG in KHS	3468	2922, 2855	1732	1672		1458, 1349	1249	1098	947, 847, 723
10% AG in 1:2 KHS:NMP	3424	2925, 2870	1756, 1732	1662	1504	1404	1299, 1111		985, 850, 656
1:2 KHS:NMP	3447	2924, 2870	1729	1664	1504	1403	1299, 1111		985, 850, 656
KHS	3464	2922, 2855	1732	1644		1458, 1349	1249	1098	947, 847, 723
AGLS-100N	3423	2928, 2868	1753, 1734	1665	1507	1406	1302	1038	656
AGLS-25M75N	3389	2927, 2873	1756, 1734	1665	1507	1406	1302	1028	813, 656
AGLS-50M50N	3339	2927, 2870	1756, 1728	1655	1508	1408	1306	1029	660
AGLS-100M	3334, 3295	2897, 2865	1725	1651	1507	1427	1313	1031	813, 661
AG-(B-LS-100N)-PM	3454, 3399	2927, 2864	1725	1665	1507	1406	1300	1026	848, 654
AG-(B-LS-100M)-PM	3333, 3302	2928, 2868	1724	1650	1507	1427	1312	1031	814, 660
B-LS-100N	3444	2927, 2860	1732	1664	1507	1406	1302	1025	847, 656
B-LS-100M	3333, 3299	2893, 2856	1732	1649	1507	1427	1313	1031	813, 661
